# Venomics across the *Bothrops neuwiedi* Species Complex Revealed a P‑III Snake Venom Metalloproteases/K49-PLA_2_ Dichotomy and a Remarkable Paraspecific Neutralization of the Brazilian Pentabothropic Antivenom

**DOI:** 10.1021/acs.jproteome.5c00933

**Published:** 2026-01-22

**Authors:** Nathália da Costa Galizio, Caroline Serino-Silva, Caroline Fabri Bittencourt Rodrigues, Daniel Rodrigues Stuginski, Marisa Maria Teixeira da Rocha, Eliana de Oliveira Serapicos, Cibele Cintia Barbarini, Roberto Baptista de Oliveira, Sávio Stefanini Sant’Anna, Kathleen Fernandes Grego, Libia Sanz, Jordi Tena-Garcés, Adolfo R. de Roodt, Juan J. Calvete, Anita Mitico Tanaka-Azevedo, Karen de Morais-Zani

**Affiliations:** † Laboratório de Fisiopatologia, 196591 Instituto Butantan, São Paulo 05503-900, Brazil; ‡ Programa de Pós-Graduação Interunidades em Biotecnologia, Universidade de São Paulo, Instituto de Pesquisas Tecnológicas, Instituto Butantan, São Paulo 05508-900, Brazil; § Laboratório de Herpetologia, Instituto Butantan, São Paulo 05503-900, Brazil; ∥ Núcleo Regional de Ofidiologia de Porto Alegre, 344048 Fundação Zoobotânica, Porto Alegre 90620-000, Brazil; ⊥ Laboratorio de Venómica Evolutiva y Traslacional, 54426 Instituto de Biomedicina de Valencia, Consejo Superior de Investigaciones Científicas, Valencia 46010, Spain; # Instituto Nacional de Producción de Biológicos, A.N.L.I.S. ″Dr. Carlos G. Malbrán″, Ministerio de Salud de la Nación, Buenos Aires C1282 AFF, Argentina; ¶ Primera Cátedra de Toxicología & Laboratorio de Toxinopatología, Centro de Patología Experimental y Aplicada, Facultad de Medicina, Universidad de Buenos Aires, Buenos Aires C1121ABG, Argentina

**Keywords:** Bothrops venom variability, venom phenotypic dichotomy, antivenomics, immunological cross-reactivity, B. neuwiedi clade

## Abstract

Snakes of the *Bothrops neuwiedi* complex are widely distributed and represent medically important species in Brazil. Here, we report compositional and functional profiles of the venom of seven species of *Bothrops neuwiedi* group: *Bothrops mattogrossensis*, *Bothrops pauloensis*, *Bothrops pubescens*, *Bothrops diporus*, *Bothrops neuwiedi*, *Bothrops marmoratus,* and *Bothrops erythromelas*. Toxin composition of individual and pooled venoms showed remarkable inter- and intraspecific variability of the relative abundance of toxins (evidenced by SDS-PAGE and RP-HPLC) and enzymatic activities (proteolytic, PLA_2_, and thrombin-like activities). *In vivo* analyses showed that *B. erythromelas* venom is the most hemorrhagic, *B. diporus* was the most lethal, and *B. pubescens* showed the highest myotoxic activity. Histopathological analysis showed that all venoms induced edema, hemorrhage, inflammatory infiltrate, and necrosis of muscle fibers. Consistent with large research evidence on the paraspecificity of various commercial antivenoms generated in Latin America, the pentabothropic antivenom produced by Instituto Butantan showed a high profile of immunoreactivity and lethality neutralization capability toward the venoms of the seven species of the *B. neuwiedi* clade. Interpreted through the prism of evolution, our data revealed a PIII-SVMP/K49-PLA_2_ compositional dichotomy and a remarkable conservation of immunological cross-reactivity across congeneric venoms throughout the 12–16 million years of *Bothrops* phylogeny.

## Graphical abstract

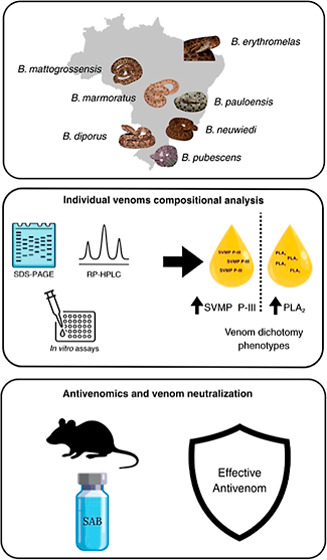

## 1 Introduction

Snakebite envenoming (SBE) is an occupational hazard and a WHO category A neglected tropical disease, affecting 4.5–5.4 million people, often young agricultural workers, living in economically depressed rural communities in tropical and subtropical regions of Africa, Asia, and Latin America.^1,2^ SBE is a “disease of poverty” that annually claims >100.000 deaths worldwide and leaves victims with permanent physical sequelae and chronic mental morbidity that affect not only the surviving victims but also their entire families, which enter a cycle of generational poverty that is difficult to break.^3^ The timely intravenous administration of safe and effective antivenoms represents the only scientifically validated treatment for snakebite envenomings.^4,5^ Since the development of the first antivenoms in Brazil in 1901, there have been antivenom manufacturers in Latin America, including Argentina, Bolivia, Brazil, Colombia, Costa Rica, Mexico, Peru, and Venezuela.^6^ Pit vipers of the genus *Bothrops* (Wagler 1824) include 48 species,^7^ which inhabit most of the ecoregions along South America, from tropical rainforests, lowland or mountainous areas, grasslands, and dry habitats.^8^

Thirty species of the genus can be found in Brazil. Between 2012 and 2021, a total of 202,604 cases of envenoming caused by *Bothrops* spp. were notified, resulting in 766 fatalities.^9^ Only in 2023, the Brazilian Notifiable Diseases Information System (SINAN) recorded 25,115 cases of snakebite resulting in 119 deaths.^10^ About 86.4% of these accidents were associated with bites by snakes of the genus *Bothrops*.^10^ The *Bothrops neuwiedi* group of species comprises medically important snakes distributed from northeastern Brazil to southern Argentina, through Bolivia, Peru, Paraguay, and Uruguay.^11^ In Brazil, with the notable exception of the Amazon region, species of the *B. neuwiedi* group occur in almost every other Brazilian ecoregion.^12^ The morphological diversity observed in the *B. neuwiedi* species complex described by Wagler (1824)^13^ was associated with meristic variations of the nominal species subdivided into 12 geographic subspecies.^14,15^ In 2008, da Silva and Rodrigues^16^ performed a comprehensive taxonomic review of the group, recognizing the long-standing 12 subspecies as seven species: *Bothrops neuwiedi*, *Bothrops diporus*, *Bothrops lutzi*, *Bothrops mattogrossensis*, *Bothrops pauloensis*, *Bothrops pubescens*, and describing the new species *Bothrops marmoratus*.^16^ Subsequently, Machado et al. (2014)^15^ revised the phylogenetic relationships within the *B. neuwiedi* complex, including *Bothrops erythromelas* as part of the taxonomic group. These authors demonstrated that the expanded *B. neuwiedi* complex represents a strongly supported monophyletic group and a sister clade of *Bothrops jararaca*.^15^ This conclusion was corroborated by Alencar et al. (2016)^17^ (Figure 1B) and Carrasco et al. (2023)^18^ (Figure 1A), who, in addition, included a new species (*Bothrops sonene* from the Peruvian Pampas del Heath, in the Bahuaja-Sonene National Park^11^) in their proposed monophyletic *B. neuwiedi* group. The *B. neuwiedi* and *B. jararaca* lineages diverged in the late Miocene (6–5 Mya),^15,17^ with subsequent Neogene/Quaternary (5–2 Mya) diversification to their present phylogeny of two major lineages within the *B. neuwiedi* clade, {*B. mattogrossensis*, *B. pauloensis*, *B. diporus*, *B. pubescens*, and *B. sonene*} and {*B. neuwiedi*, *B. marmoratus*, *B. lutzi*, and *B. erythromelas*}.^15,17,18^

**Figure 1:**
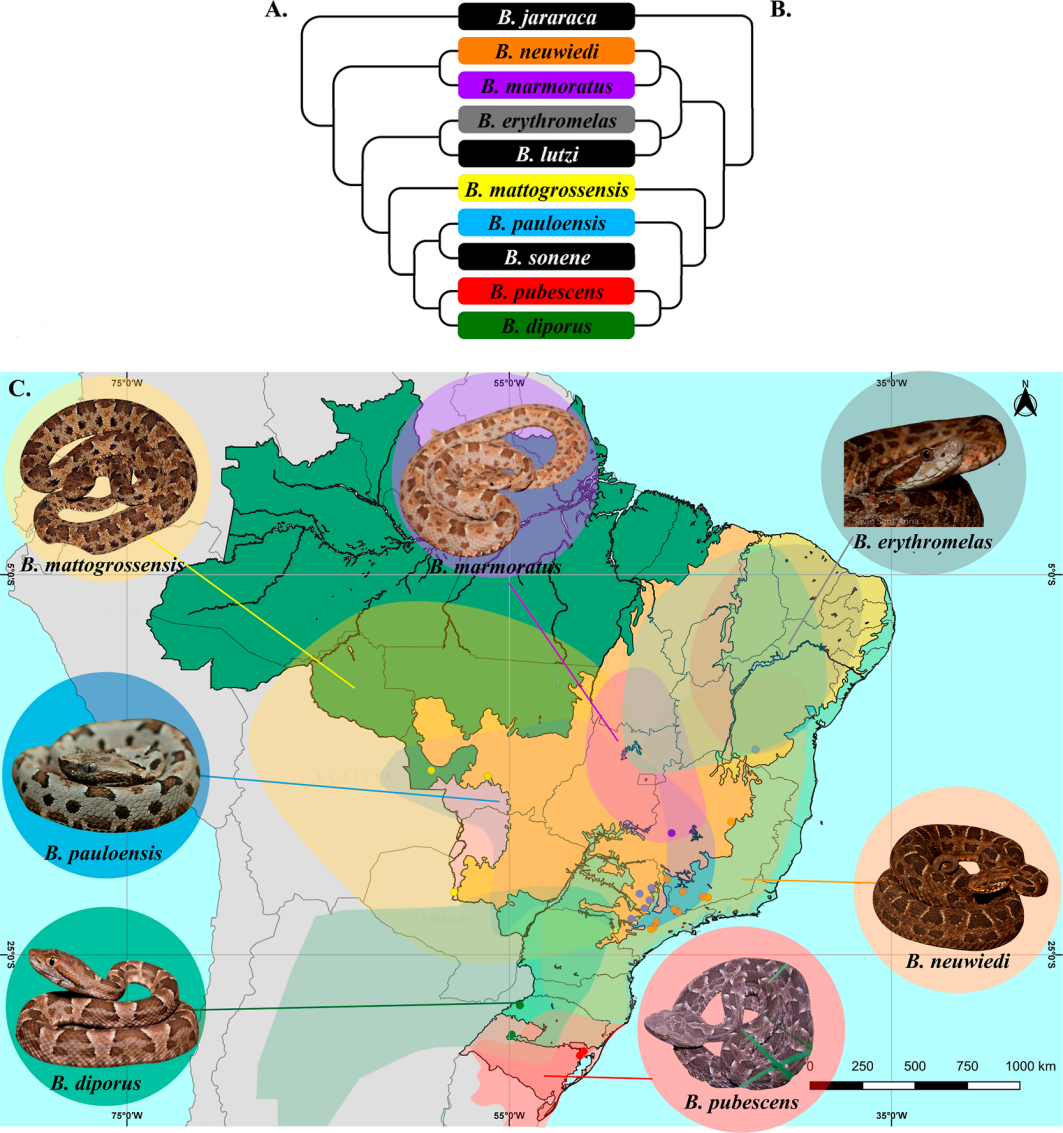
Phylogenetic relationship of *Bothrops neuwiedi* complex. (A) Phylogenetic tree based on Carrasco et al. (2023).^18^ (B) Phylogenetic tree based on Alencar et al. (2016).^17^ (C) Geographic location of the specimens used in this study was plotted on a biome map. Biomes represented in (1) dark green color: Amazon rainforest; (2) light orange: Cerrado; (3) light purple: Pantanal; (4) light brown: Caatinga; (5) light green: Atlantic Forest; and (6) purple: Pampas. Points plotted in (a) yellow: *B. mattogrossensis*, (b) gray: *B. erythromelas*; (c) purple: *B. marmoratus*; (d) blue: *B. pauloensis*; (e) orange: *B. neuwiedi*; (f) green: *B. diporus*; and (g) red: *B. pubescens*.

Despite the medical importance of the venoms produced by snakes of the *B. neuwiedi* species group in their respective geographic ranges (see Figure 2 in Carrasco et al., 2019 ^11^), their toxin composition and functional profile have been only reported for *B. diporus* from central (Córdoba), northern (Formosa), and northeastern (Catamarca, Entre Rios, and Misiones) Argentina;^19,20^ *B. pauloensis* from São Paulo, Brazil;^21,22^ *B. pubescens* from the Brazilian State of Rio Grande do Sul;^23^ and *B. erythromelas* from the Caatinga ecoregion in northeast Brazil.^24^ Other studies did not specify the subspecies sampled or employed a pool of venoms from different subspecies.^25–27^ On the other hand, the immunization mixture used by Instituto Butantan, the institution responsible for the production of about 90% of the serums and vaccines in Brazil,^28^ to produce the equine bothropic polyvalent F­(ab’)_2_ antivenom (soro antibotrópico—SAB) comprises venoms from *B. jararaca* (50%, mainly from the SE phylogroup^29^), *B. alternatus* (12.5%), *B. jararacussu* (12.5%), *B. moojeni* (12.5%), and the *B. neuwiedi* group (12.5%, variable relative amounts of currently recognized species).^30,31^

**Figure 2:**
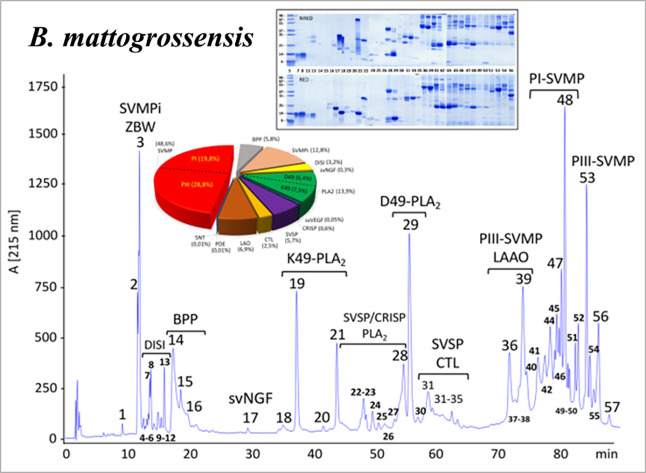
Venom proteome of *Bothrops mattogrossensis*. The venom proteome was assessed through a bottom-up venomics workflow that includes the decomplexation and quantification of the venom components by reverse-phase (RP) chromatography and SDS-PAGE analysis of the chromatographic fractions, and downstream mass spectrometric identification of the venom toxins. SVMPi, tripeptide inhibitor of snake venom metalloproteinases (ZBW); DISI, disintegrin; BPP, bradykinin-potentiating peptides; svVGF, snake venom vascular growth factor; K49-PLA2, K49-phospholipase A_2_ homologues; D49-PLA2, D49-phospholipase A_2_; SVSP, snake venom serine proteinase; CRISP, cysteine-rich secretory protein; CTL, C-type lectin-like protein; PI-SVMP, snake venom metalloproteinase from class I; PIII-SVMP, snake venom metalloproteinase from class III; and LAAO, l-amino acid oxidase. Details of the individual proteins are shown in Supporting Information, S2.

Defining the phylogeographic boundaries of an antivenom’s effectiveness has implications for optimizing its clinical use. There is a large body of evidence in the literature on the paraspecific effectiveness of bothropic antivenoms produced in Latin American countries in the neutralization of congeneric venoms, e.g., Moura-da-Silva et al. (1989);^32^ de Roodt et al., 1998;^33^ Otero-Patiño et al., 2002;^34^ Rojas et al., 2005;^35^ Queiroz et al., 2008;^36^ Gutiérrez, 2009;^37^ Segura et al., 2010;^38^ Dias da Silva and Tambourgi, 2011;^39^ Sousa et al., 2013;^40^ de Roodt et al., 2014;^41^ and Mora-Obando et al., 2021.^42^ Notwithstanding reported intra- and interspecific bothropic venom variability attributed to ontogenetic shift,^22,43–47^ gender,^48,49^ and geographic origin^29,50,51^ that could had affected antivenom efficacy, there is remarkable para-specificity exhibited by antivenoms generated against immunization mixtures that included venoms from phylogenetic distant *Bothrops* species, which may be ascribed to a large conservation of immunoreactive epitopes on venom toxins across much of the natural history of *Bothrops*, a genus that has its roots in South America during the middle Miocene, (14.07 (CI95:16.37–11.75) Mya^17,52^ ). Here, we compare the structural and functional venomics profiles of *B. mattogrossensis*, *B. pauloensis*, *B. pubescens*, *B. diporus*, *B. neuwiedi*, *B. marmoratus,* and *B. erythromelas*. Additionally, we report a comprehensive toxin-resolved antivenomics analysis and the *in vivo* capability of the pentabothropic antivenom produced by Instituto Butantan to neutralize the lethality of *B. mattogrossensis*, *B. neuwiedi*, *B. pubescens*, and *B. marmoratus* in mice.

## 2 Experimental Section

### 2.1 Snakes, Venoms, and Antivenom

This study was registered with the Brazilian National System for the Management of Genetic Patrimony and Associated Traditional Knowledge (SISGEN, registration no. AC75E7B). Venoms of *B. neuwiedi*, *B. marmoratus*, *B. pauloensis*, *B. mattogrossensis,* and *B. erythromelas* were obtained from adult snakes kept in captivity at the Laboratory of Herpetology, Instituto Butantan, São Paulo, Brazil. Venoms of *B. diporus* and *B. pubescens* were obtained from adult snakes kept in captivity at the Ncleo Regional de Ofidiologia de Porto Alegre, Fundação Zoobotânica, Porto Alegre, Brazil. Animals were maintained under controlled temperature, humidity, and light/dark cycle (12:12). The snakes were fed on rodents (*Mus musculus* and/or *Rattus norvegicus*) once a month.^53^ The geographic origin and sex from all the snakes used in this work are disclosed in Supporting Information, S1 and Figure 1C. Due to specimens availability, the *B. marmoratus* venom sample comprised venom from two individuals and their six offspring (adults). Unfortunately, it was not possible to include *B. lutzi* and *B. sonene* venoms in the present study due to a lack of availability. Venoms were centrifuged for 15 min at 1,700*g*, lyophilized, and stored at −20 °C until use. Venoms were evaluated individually by *in vitro* tests; however, for *in vivo* assays, venom pools composed of equal amounts of lyophilized individual species venoms were used. Protein concentration was determined on individual and pooled venoms according to the method described by Bradford,^54^ using the Bio-Rad Protein Assay reagent and bovine serum albumin as standard. All samples were analyzed in triplicate.

The bothropic polyvalent F­(ab’)_2_ antivenom (soro antibotrópico—SAB—batch 220015; expiration date 07/2025) used in the immunorecognition assays was provided by Instituto Butantan (São Paulo, Brazil). This antivenom was produced by hyperimmunization of horses against a pool of four *Bothrops* species venoms, namely, *B. jararaca* (50%), *B. alternatus* (12.5%), *B. jararacussu* (12.5%), *B. moojeni* (12.5%), and the *B. neuwiedi* complex (*B. neuwiedi*, *B. pauloensis*, *B. mattogrossensis,* and *B marmoratus*, 12.5%). The final formulation consists of purified F­(ab’)_2_ fragments generated by digestion with pepsin of ammonium sulfate-precipitated IgG molecules. A vial of SAB contained 46.45 mg/L of F­(ab’)_2_ and a nominal neutralization capacity of 50 mg of *B. jararaca* venom (the reference venom for assessing the bothropic antivenom potency in Brazil).

### 2.2 Compositional Analysis of *B. neuwiedi* Clade Snake Venoms

The toxin composition of the venoms of *B. neuwiedi*, *B. marmoratus*, *B. pauloensis*, *B. mattogrossensis*, *B. erythromelas*, *B. diporus,* and *B. pubescens* was analyzed by SDS-PAGE (Laemmli, 1970)^55^ under both reducing and nonreducing conditions in 15% polyacrylamide gels and visualized by Coomassie Brilliant Blue G250 (GE Healthcare) staining. The venom proteomes of these venoms were analyzed also by RP HPLC decomplexation as described (Gay et al.^19^). Briefly, 2 mg of crude, lyophilized venom was dissolved in 200 μL of 5% acetonitrile in water containing 0.1% trifluoroacetic acid (TFA), centrifuged, and submitted to RP HPLC separation using a Teknokroma Europa Protein 300C18 (0.4 cm × 25 cm, 5 mm particle size, 300 Å pore size) column and an LC 1100 High Pressure Gradient System (Agilent Technologies, Santa Clara, CA, USA). The flow-rate was set to 1 mL/min, and the column was developed with a linear gradient of 0.1% TFA in water (solution A) and acetonitrile (solution B) using the following column elution conditions: isocratically (5% B) for 5 min, followed by 5–25% B for 10 min, 25–45% B for 60 min, and 45%–70% for 10 min. Protein detection was carried out at 215 nm. Fractions were collected manually, dried in a vacuum centrifuge (Savant), redissolved in water, and submitted to SDS-PAGE analysis under reducing and nonreducing conditions. Protein classes were assigned to chromatographic peaks by similarity to elution time and electrophoretic band composition of *B. neuwiedi* clade venoms reported in the literature, *B. pauloensis*,^21^ *B. diporus*,^19^ *B. erythromelas*,^24^ *B. pubescens*,^23^ and *B. mattogrossensis* (this work). The relative abundances of the protein families present in the venoms were calculated as the ratio of the sum of the percentages of the individual proteins from the same toxin family to the total area of venom protein peaks in the RP chromatogram and graphed as pie charts.

### 2.3 Venomic Analysis of *B. mattogrossensis* Venom Proteome

The proteome of *B. mattogrossensis* venom was characterized through the bottom-up snake venomics workflow described in Eichberg et al. (2015).^56^ To this end, protein bands excised from Coomassie Brilliant Blue-stained SDS-PAGE gels of the chromatographic fractions collected through RP-HPLC venom decomplexation were subjected to automated in-gel reduction (10 mM dithiothreitol) and alkylation (50 mM iodoacetamide), followed by overnight sequencing-grade trypsin digestion (66 ng/μL in 25 mM ammonium bicarbonate, 10% acetonitrile; 0.25 μg/sample) in an automated processor (ProGest Protein Digestion Workstation, Genomic Solution Ltd., Cambridgeshire, UK). The tryptic digests were dried in a SpeedVac (SavantTM, Thermo Scientific Inc., West Palm Beach, FL, USA), redissolved in 15 μL of 0.1% formic acid in water, and submitted to LC–MS/MS. To this end, tryptic peptides were separated by nano-Acquity UltraPerformance LC using a BEH130C18 (100 μm × 100 mm, 1.7 μm particle size) column in-line with a SYNAPT G2 High-Definition Mass Spectrometry System (Waters Corp. Milford Massachusetts, USA). The flow rate was set to 0.6 μL/min, and the column was developed with a linear gradient of 0.1% formic acid in water (solution A) and 0.1% formic acid in acetonitrile (solution B), isocratically 1% B for 1 min, followed by 1% to 12% B for 1 min, 12% to 40% B for 15 min, and 40% to 85% B for 2 min. Doubly and triply charged ions were selected for collision-induced dissociation MS/MS. Fragmentation spectra were interpreted (a) manually (de novo sequencing); (b) using the online form of the MASCOT program at http://www.matrixscience.com against the NCBI nonredundant database; and (c) processed in Waters Corporation’s ProteinLynx Global SERVER 2013 version 2.5.2 (with Expression version 2.0) against the species-specific venom gland cDNA-derived toxin sequences. MS/MS mass tolerance was set to 0.6 Da. Carbamidomethyl cysteine and oxidation of methionine were selected as fixed and variable modifications, respectively. Amino acid sequence similarity searches were performed against the available databanks using the BLAST program implemented in the WU-BLAST2 search engine at http://www.bork.embl-heidelberg.de.

The relative abundances of the chromatographic peaks obtained by RP HPLC fractionation of the whole venom were calculated as “% of total peptide bond concentration in the peak” by dividing the peak area by the total area of the chromatogram.^56^ For chromatographic peaks containing single components (as judged by SDS-PAGE and/or MS), this figure is a good estimate of the % by weight (g/100 g) of the pure venom component.^57^ When more than one venom protein was present in a RP fraction, their proportions (% of total protein band area) were estimated by densitometry of Coomassie-stained SDS-polyacrylamide gels using MetaMorph Image Analysis Software (Molecular Devices). Conversely, the relative abundances of different proteins contained in the same SDS-PAGE band were estimated based on the relative ion intensities of the three most abundant peptide ions associated with each protein by MS/MS analysis. The relative abundances of the protein families present in the venom were calculated as the ratio of the sum of the percentages of the individual proteins from the same toxin family to the total area of the venom protein peaks in the RP chromatogram.

The mass spectrometry proteomics data have been deposited to the ProteomeXchange Consortium^58^ via the PRIDE^59^ partner repository with the data set identifier PXD066646.

### 2.4 *In Vitro* Functional Analysis

#### 2.4.1 Proteolytic Activity on Collagen

Collagenolytic activity was determined as described by Váchová and Moravcová (1993)^60^ and modified by Antunes et al. (2010),^61^ using azocoll as substrate. Briefly, 6.25 μg of venom was incubated with 50 μL of 5 mg/mL azocoll (Sigma) solution, both solubilized in Tyrode buffer (137 mM NaCl, 2.7 mM KCl, 3.0 mM NaH_2_PO_4_, 10 mM *N*-(2-hydroxyethyl)­piperazine-*N*′-ethanesulfonic acid, 5.6 mM dextrose, 1 mM MgCl_2_, 2 mM CaCl_2_, pH 7.4) for 1 h at 37 °C in a thermoshaker (Kasvi), with constant stirring speed set at 1,200 rpm. The reaction was stopped by placing samples on ice. After centrifugation for 3 min at 5,000*g*, the absorbance of the supernatant (200 μL) was measured at 540 nm with SpectraMax i3 microplate reader (Molecular Devices). One unit of activity was defined as the amount of venom that causes an increase of 0.003 units of absorbance, and the specific activity was expressed as U/min/mg of venom.^61^ All samples were assayed in triplicate. Data were expressed as the mean ± SD.

#### 2.4.2 Phospholipase A_2_ Activity

Phospholipase A_2_ (PLA_2_) activity was determined based on the method described by Holzer and Mackessy.^62^ Twenty microgram of venom in 40 μL in 0.85% saline was mixed with 200 μL of 10 mM Tris–HCl, 10 mM CaCl_2_, 0.1 M NaCl, pH 8.0 in a 96-well microplate. Then, 20 μL of the monodisperse synthetic substrate 4-nitro-3-octanoyloxy-benzoic acid (4-NOBA) (4.16 mM in acetonitrile) was added to a final concentration of 0.32 mM. After incubation for 60 min at 37 °C, the absorbances were recorded at 425 nm with a SpectraMax i3 microplate reader (Molecular Devices). It was assumed that a change in absorbance of 0.01 is equivalent to 25.8 nmoles of chromophore release.^62^ One unit of PLA_2_ activity corresponded to 1 nM released chromophore. Specific phospholipasic activity was expressed as nmoles of chromophore/min/mg of venom. All samples were assayed in triplicates, and data were expressed as mean ± SD.

#### 2.4.3 Thrombin-Like Activity on Chromogenic Substrate

The chromogenic substrate S-2238 (Chromogenix) was used to assess the thrombin-like activity of serine proteases according to the manufacturer’s recommendations, with some modifications. Five microliter of 1 mg/mL venom in 0.85% saline was incubated with 10 μL of chromogenic substrate S-2238 (4 mM) and 90 μL of 50 mM Tris pH 8.0 at 37 °C for 5 min. The reaction was stopped by the addition of 90 μL of 20% acetic acid, and the absorbance values were measured at 405 nm with SpectraMax i3 microplate reader (Molecular Devices). Bovine thrombin (2 U/mL; Roche) was used as a positive control. All samples were assayed in triplicate, and data were expressed as mean ± SD.

### 2.5 *In Vivo* Biological Activity Assays

Experiments using mice were approved by the Ethics Committee for Animal Use from Butantan Institute, São Paulo, Brazil (protocols 3474090218 and 9567060824), under the guidelines of the Brazilian National Council for Control of Animal Experimentation, in agreement with the International Guiding Principles for Biomedical Research Involving Animals^63^ and the ARRIVE guidelines for the Report for *in vivo* experiments.^64^

#### 2.5.1 Lethal Dose 50%

The LD_50_ of the venom pools was determined by Probit analysis^65^ using the StatPlus software version 5.8.4.3 (AnalystSoft). To this end, different doses of venom (ranging from 23 to 230 μg) solubilized in 500 μL of sterile 0.85% saline were injected intraperitoneally in 5 male Swiss mice (18–22 g) per dose group, and deaths were registered at 48 h.

#### 2.5.2 Minimum Hemorrhagic Dose

For the determination of minimum hemorrhagic dose (MHD), the method described by Kondo et al. (1960)^66^ modified by Gutiérrez et al. (1985)^67^ was used. Increasing doses of venom pools (1.25–15 μg/mouse) in 50 μL of sterile 0.85% saline solution were injected by intradermal route into the abdominal skin of groups of 5 male Swiss mice (18–22 g). After 3 h, the animals were euthanized, and the abdominal skin was removed, photographed, and digitalized. The hemorrhagic area was obtained using the ImageJ software version 1.51j8. The MHD was defined as the amount of venom that produces hemorrhage with a mean area of 1 cm^2^ after 3 h of venom injection, and results are expressed as mean ± SD.

#### 2.5.3 Edematogenic Activity

The edematogenic activity of venom pools was evaluated in groups of 5 male Swiss mice weighing 18–22 g. Edema was induced by the injection of 1 μg of venom into the subplantar tissue of the mouse right hind paw.^68^ The contralateral paw received the same volume of a 0.85% saline solution. Paw edema was determined by measuring paw thickness using a digital caliper at 0 (time before subplantar injection of snake venoms or sterile saline), 1, 2, 4, 6, and 24 h post injection. Results were calculated as the difference in the thickness of both paws, and edema was expressed as the percentage increase in paw thickness. Data were expressed as mean ± SD.

#### 2.5.4 Myotoxicity

The myotoxic activity of venoms was determined as described by Segura et al. (2017)^69^ and Gutiérrez et al. (2008).^70^ Fifty microgram of venom (1 mg/mL in 50 μL of 0.85% saline) was injected intramuscularly (i.m.) in the right *gastrocnemius* muscle of 5 male Swiss mice weighing 18–22 g. Mice in the control group received 50 μL of 0.85% saline only. Three hours after the injection, the mice were bled from the tail, and blood was collected in heparinized capillaries. The activity of the creatine kinase enzyme was quantified using the CK NAC UV kit (Bioclin) and expressed as mean ± SD in units per liter (U/L).

Additionally, after blood collection, animals were euthanized, and the injected gastrocnemius muscles were dissected and immediately placed in a 10% formalin solution. After routine processing, tissue samples were embedded in paraffin, and 5 μm sections were obtained and stained with hematoxylin and eosin for histological assessment.

### 2.6 Immunorecognition Profile of the Brazilian Pentabothropic F­(ab’)_2_ Antivenom

#### 2.6.1 Immunoaffinity Chromatography-Based Antivenomics

The third-generation antivenomics (3GA) protocol described by Pla et al. (2017)^71^ and Calvete et al. (2018)^72^ was applied to assess the immunoreactivity of the Brazilian bothropic polyvalent F­(ab’)_2_ antivenom (SAB) toward the venoms of *B. mattogrossensis*, *B. neuwiedi*, *B. pubescens,* and *B. marmoratus*. The antivenom chromatographic matrix was prepared in a batch of 3 mL of CNBr-activated Sepharose 4B matrix (GE Healthcare, refs 17-0430-01). Before antivenom coupling, the CNBr-activated matrix batch was washed with 10–15 matrix volumes of cold 1 mM HCl and two matrix volumes of coupling buffer (0.2 M NaHCO_3_, 0.5 M NaCl, pH 8.3) to adjust the pH of the matrix between 7.0 and 8.0. The washed matrix was incubated for 4h at room temperature with 100 mg of antivenom, which had been extensively dialyzed against deionized water to remove nonprotein substances, i.e., excipients and stabilizers, lyophilized, and solubilized in coupling buffer at approximately 85 mg/mL. The supernatant containing nonbound F­(ab’)_2_ molecules was decanted, the affinity matrix was washed with 15x matrix volumes of cold 1 mM HCl, followed by two matrix volumes of coupling buffer to adjust the pH of the column to 8.0–9.0, and the protein concentration of coupled antivenom was estimated spectrophotometrically from the difference of absorbance at 280 nm in a 1 cm cuvette before and after the coupling of the antivenom using an extinction coefficient (ε^0.1%^) of 1.36 (mg/mL)^−1^ cm^–1^.^73^ The immunoaffinity matrix thus prepared contained 70 mg of antivenom/mL. Nonreacted groups were then blocked with a matrix volume of 0.1 M Tris–HCl, pH 8.0, at 4 °C overnight using an orbital shaker. The affinity matrices were washed alternately at high and low pH, with three volumes of 0.1 M acetate buffer, 0.5 M NaCl, pH 4.0–5.0 and three volumes of 0.1 M Tris–HCl buffer, pH 8.5. This treatment was repeated 6 times.

Before incubation with the crude venoms, the immunoaffinity columns were equilibrated with five matrix volumes of binding buffer (phosphate-buffered saline, PBS). For the immunoaffinity assays, sets of seven columns each containing 7 mg of F­(ab’)_2_ immobilized onto 350 μL matrix were incubated with 100–3600 μg total proteins of the crude venoms dissolved in 1/2 matrix volume of PBS, and incubated for 1 h at 25 °C using an orbital shaker. As specificity controls, 350 μL of CNBr-activated Sepharose 4B matrix, with or without 7 mg of immobilized control (naïve) IgGs, were incubated with 3600 μg of the corresponding venom and run in parallel to the immunoaffinity columns. Thereafter, antivenom and the specificity (naïve IgG) and mock matrix control columns were washed 5 times with PBS and eluted with 5 matrix volumes of elution buffer (0.1 M glycine-HCl pH 2.0), and the pH was neutralized with neutralization buffer (1 M Tris–HCl pH 9.0). The crude venom and the nonretained and retained fractions recovered from the affinity chromatography experiments were analyzed by RP HPLC using a Discovery BIO Wide Pore C18 (15 cm × 2.1 mm, 3 μm particle size, 300 Å pore size) column and an Agilent LC 1100 High-Pressure Gradient System equipped with DAD detector and microautosampler. The flow rate was set to 0.4 mL/min, and the column was developed with a linear gradient of 0.1% TFA in water (solution A) and 0.1% TFA in acetonitrile (solution B): isocratically (5% B) for 1 min, followed by 5–25% B for 5 min, 25–45% B for 35 min, and 45–70% for 5 min. Protein detection was carried out at 214 nm with a reference wavelength of 400 nm. The fractions (%) of immunocaptured and nonimmunocaptured Toxin “i” (Tx_i_) were estimated as the relative ratios of the chromatographic areas of the same protein recovered in the nonretained (NRi) and retained (Ri) affinity chromatography fractions using the equation %Ri = [(Ri/(Ri + NRi)) x 100], and the μg of Tx_i_ recovered from each species-specific affinity column was calculated as (% Ri/100) × [(%Tx_i_ in the venom proteome × (μg Tx_i_ incubated in the affinity column/100)]. The maximal binding capacity of 7 mg of the Brazilian pentabothropic F­(ab’)_2_ antivenom was estimated as the sum of the maximal binding for each venom toxin component by the set of species-specific affinity columns comprising a 3GA experiment. The calculations are illustrated in Supporting Information, S7–S10.

### 2.7 *In Vivo* Neutralization of Venom Lethality by the Brazilian pentabothropic F­(ab’)_2_ *Antivenom*

The capacity of the pentabothropic antivenom produced by Instituto Butantan to neutralize the lethal activity of venoms from the *B. neuwiedi* complex was assessed by intraperitoneal injection of 500 μL of a solution containing 5 × LD_50_, which was previously incubated for 30 min at 37 °C with different dilutions of the antivenom, to groups of 5 male Swiss mice (18–22 g). Deaths were recorded after 48 h, and the median effective dose (ED_50_) was estimated by Probit analysis^65^ using the StatPlus software version 5.8.4.3 (AnalystSoft). The potency (i.e., the amount of venom (mg) neutralized per 1 mL of antivenom) was calculated as *P* = [(*n* – 1)/ED_50_] × LD_50_, where “*n*” is the number of LD_50_s used as challenge dose to determine the ED_50_. “*n* – 1” is used because, at the end point of the neutralization assay, the activity of one LD_50_ remains unneutralized, causing the death of 50% of mice.^74,75^ Potency enables an estimation of the amount of antivenom required to provide complete neutralization of a given quantity of venom. Potency and ED_50_ are related by the formula

$$P=\frac{{ED}_{50}}{\left(\frac{n}{n-1}\right)}$$

### 2.8 Statistical Analysis

The Shapiro–Wilk’s test for normality and Levene’s test for homogeneity of variances were conducted. Following results were statistically analyzed using one-way analysis of variance (ANOVA), followed by the Tukey test. Differences with *p* < 0.05 were considered statistically significant. For edematogenic activity, repeated measures ANOVA were performed. General Linear Models were calculated to establish correlations between MHD and LD_50,_ and between K49-PLA_2_ and P-III snake venom metalloproteases (SVMP) HPLC peaks. Due to the complexity of representing the statistical results in the figures, a comparative statistical table for each venom biological effect tested is displayed in Supporting Information, S5. Statistical analyses were performed using GraphPad Prism software (version 8) and R software (packages car, ggplot2).

## 3 Results and Discussion

### 3.1 Bottom-Up Proteomic Analysis of the Toxin Arsenal of *Bothrops mattogrossensis* Venom

The venom proteome of *B. mattogrossensis* was unveiled through our bottom-up venomics workflow that includes a first step of decomplexation and quantification of the venom components by RP chromatography and SDS-PAGE analysis of the chromatographic fractions and downstream mass spectrometric identification of the venom toxins^56^ (Figure 2, Supporting Information, S2). The RP chromatographic profile of *B. mattogrossensis* venom displays the “*Bothrops venom pattern*”, which resembles that of other *Bothrops* species.^40^ Roughly, it is qualitatively characterized by the sequential elution of tripeptide inhibitors of snake venom metalloproteinases (SVMPi), medium-sized disintegrins (DISI), bradykinin-potentiating peptides, BPP (BPPs), K49-phospholipase A_2_ homologues (K49-PLA_2_), cysteine-rich secretory proteins (CRISP), catalytically active D49-phospholipase A_2_ proteins (D49-PLA_2_), snake venom serine proteinases (SVSP) + C-type lectin-like (CTL) proteins, L-amino acid oxidase(LAAO) + SVMP, and in the very last fractions of the chromatographic elution PI- and PIII-SVMPs (Figure 2; Supporting Information, S2). In quantitative terms, *B. mattogrossensis* venom toxin proteome is composed predominantly by Zn^2+^-SVMP of classes PIII (28.8% of the venom proteome) and PI (19.8%) and the endogenous tripeptides (pyroGlu-(Lys/Gln/Gln)-Trp) (ZBW, 12.8%), with less contribution from catalytically active (D49, 6.4%) and myotoxic (K49, 7.5%) PLA_2_, LAAO (6.9%), BPP (5.8%), SVSP (5.7%), DISI (3.2%), and CTL (2.5%) molecules. Other toxins, e.g., snake venom nerve and vascular endothelial growth factors, svNGF and svVEGF, respectively, CRISP, phosphodiesterase, and 5′-nucleotidase (5NT) were identified in very low (<1%) abundance. This toxin arsenal may account for the hemotoxic and proteolytic activities underlying the hemorrhagic, coagulopathic, defibrinogenating, myotoxic, dermonecrotic, and nephrotoxic effects commonly described in bothropic envenomings.^76–82^ On the other hand, the tripeptide ZBW accounts for 12.8% (28.8 × 10^–4^ mmoles %) of the total RP-HPLC chromatogram area. ZBW, which are released into the venom proteome by the proteolytic processing of a common precursor with BPPs,^83,84^ have been characterized as weak reversible endogenous inhibitors (IC_50_ in the range of 0.15–0.95 mM) of the fibrinogenolytic activity of SVMP^85^ under physiological conditions.^86^ At the concentration found in *B. mattogrossensis* venom, tripeptide ZBW represents 89.8% of all the venom polypeptide molecules. The 20.4 [SVMPi]: 1 [(PI + PIII)-SVMP] molar ratio may protect glandular tissues and venom factors from the proteolytic activity of SVMPs stored at high concentration in an inactive but competent state for many months in the lumen of the venom gland of many Viperidae snakes.^87–89^

### 3.2 Comparative SDS-PAGE and RP-HPLC Profiling Unveil Distinct Compositional Patterns in *B. neuwiedi* Clade Venoms

SDS-PAGE analysis of the protein profiles of individual and pooled venoms from *B. mattogrossensis*, *B. pauloensis*, *B. pubescens*, *B. diporus*, *B. neuwiedi*, *B. marmoratus,* and *B. erythromelas* revealed intra- and interspecific venom variability in their band composition and intensity (Figure 3). RP chromatographic profiling showed that, notwithstanding the electrophoretic variability between conspecific venoms and among venoms of different species of the same evolutionary clade (Figure 1), all the venoms of the *B. neuwiedi* complex species exhibited the highly conserved qualitative *Bothrops* venom pattern (Figure 4, Supporting Information, S3 and S4). Toxin class assignment to chromatographic peaks, inferred by similarity to elution time and electrophoretic band composition of *B. neuwiedi* clade venoms reported in the literature, including *B. pauloensis*,^21^ *B. diporus*,^19^ *B. erythromelas*,^24^ *B. pubescens*,^23^ *B. mattogrossensis* (this work), and venom proteomes of *Bothrops asper* lineages from the Pacific sides of Ecuador and southwestern Colombia,^90^ showed that *B. neuwiedi* clade venoms are predominantly comprised by (PI + PIII) SVMP and (K49 + D49) PLA_2_ molecules and, to a minor extent SVSP, DISI, and LAAO (Figures 2 and 4; Supporting Information, S3 and S4). Relative toxin abundances calculated from RP-HPLC chromatograms monitored at 215 nm correspond to % by weight (g toxin class/100 g of venom).^57^ Expressing the compositional data in millimoles %, all the *B. neuwiedi* clade venoms contained high (18 ± 6) SVMPi/SVMP molar ratios. Also, all the *B. neuwiedi* clade venoms analyzed showed a higher concentration of PI-SVMP than PIII-SVMPs in the context of three distinct compositional patterns characterized by their K49-PLA_2_/D49-PLA_2_ mmol % ratios: (i) *B. diporus*, *B. neuwiedi*, *B. pubescens*, and *B. pauloensis* expressed the highest concentration of K49-PLA_2_ and lowest content of PIII-SVMPs; (ii) the highest concentration of D49-PLA_2_, lack of K49-PLA_2_s, and similar low concentration of PIII- and PI-SVMPs (*B. erythromelas*); and (iii) a similar proportions of K49- and D49-PLA_2_s (*B. mattogrossensis* and *B. marmoratus* venoms) (Table 1, Supporting Information, S4).

**Figure 3:**
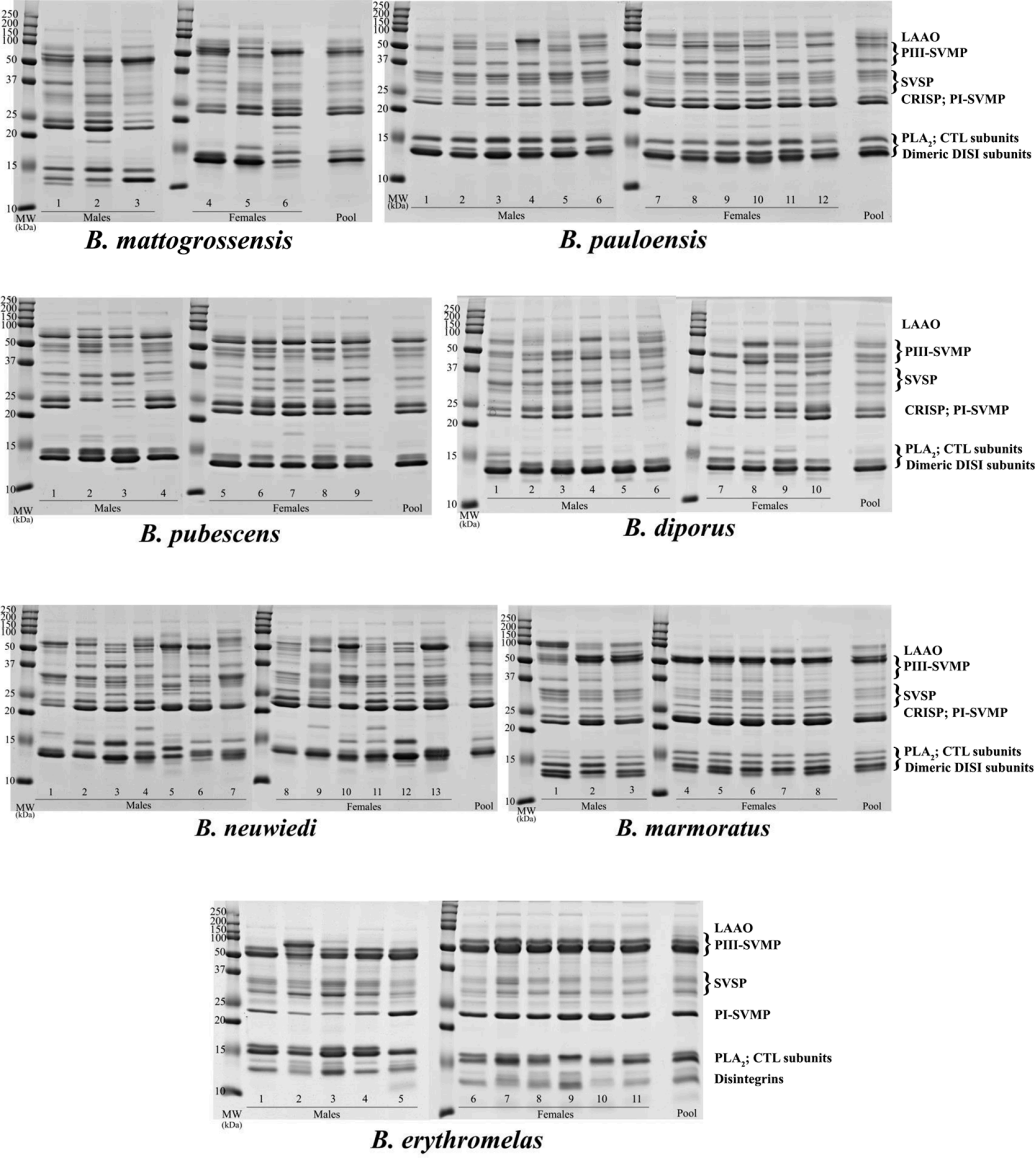
Electrophoretic profiles of *B. neuwiedi* clade venoms. Individual and pooled venoms (20 μg) were subjected to 15% SDS-PAGE, under reducing conditions, and proteins were stained using Coomassie Blue G (GE Healthcare). MW: molecular weight marker (Dual Color Precision Plus Protein Standards—BioRad). Numbers indicated the specimen according to Supporting Information, S1. Toxin class assignment was inferred by similarity to electrophoretic band composition of *B. neuwiedi* clade venoms reported in the literature. PLA2, phospholipase A_2_; SVSP, snake venom serine proteinase; CRISP, cysteine-rich secretory protein; CTL, C-type lectin-like protein; PI-SVMP, snake venom metalloproteinase from class I; PIII-SVMP, snake venom metalloproteinase from class III; and LAAO, l-amino acid oxidase.

**Figure 4:**
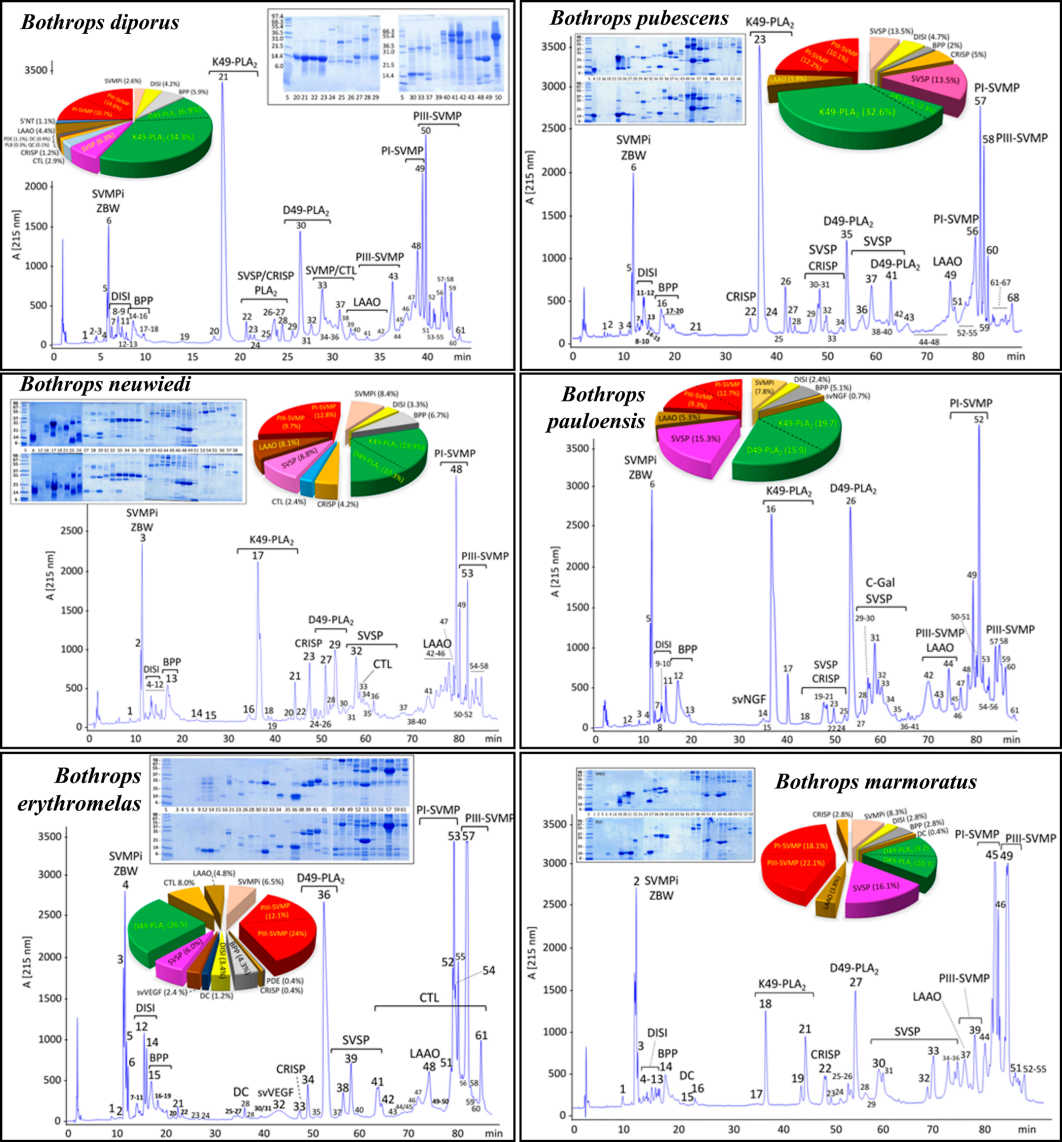
Reversed-phase chromatographic profiles and SDS-PAGE from the extracted chromatographic peaks of venoms from *B. neuwiedi* clade. Venom samples of 2 mg were subjected to RP-HPLC on a C18 column in a LC 1100 High Pressure Gradient System (Agilent Technologies). The flow rate was set at 1 mL/min, and the column was developed with a linear gradient of 0.1% TFA (solution A) and acetonitrile (solution B), as described in experimental section. Fractions were collected and submitted to SDS-PAGE analysis under reducing and nonreducing conditions. Relative abundance of protein families was calculated as the ratio of the sum of percentages of the individual proteins from the same toxin family to the total area of venom protein peaks and graphed as pie charts (Supporting Information, S4). SVMPi, tripeptide inhibitor of snake venom metalloproteinases (ZBW); DISI, disintegrin; BPP, bradykinin-potentiating peptides; svVGF, snake venom vascular growth factor; K49-PLA2, K49-phospholipase A_2_ homologues; D49-PLA2, D49-phospholipase A_2_ ; SVSP, snake venom serine proteinase; CRISP, cysteine-rich secretory protein; CTL, C-type lectin-like protein; PI-SVMP, snake venom metalloproteinase from class I; PIII-SVMP, snake venom metalloproteinase from class III; and LAAO, l-amino acid oxidase.

**Table 1:** Relative Abundances (g/100 g Venom) and Percentages of Toxin Molecules (mmoles %) of Major Toxin Classes in *Bothrops neuwiedi* Clade Venoms^a^

		*B. mattogrossensis*		*B. diporus*		*B. neuwiedi*		*B. erythromelas*		*B. pubescens*		*B. pauloensis*		*B. marmoratus*	
	mass [Da]	% (g/100 g)	mmoles %	% (g/100 g)	mmoles %	% (g/100 g)	mmoles %	% (g/100 g)	mmoles %	% (g/100 g)	mmoles %	% (g/100 g)	mmoles %	% (g/100 g)	mmoles %
K49-PLA_2_	1.40 × 10^4^	7.5	0.536	34.3	**2.450**	19.9	**1.421**			32.6	**2.329**	19.7	**1.407**	10.7	0.764
D49-PLA_2_	1.40 × 10^4^	6.4	0.457	6.9	0.493	10.3	0.736	26.5	1.893	2.3	0.164	15.9	1.136	9.2	0.657
PI-SVMP	2.30 × 10^4^	19.8	0.861	10.7	0.465	12.8	0.557	12.1	0.526	12.2	0.530	12.7	0.552	18.1	0.787
PIII-SVMP	5.20 × 10^4^	28.8	0.554	14.3	**0.275**	9.7	**0.187**	24.0	0.462	10.1	**0.194**	9.3	**0.179**	22.1	0.425
SVSP	2.80 × 10^4^	5.7	0.204	6.3	0.225	8.8	0.314	6.0	0.214	13.5	0.482	15.3	0.546	16.1	0.575
DISI	7.50 × 10^3^	3.2	0.427	4.2	0.560	3.3	0.440	3.4	0.453	4.7	0.627	2.4	0.320	2.8	0.373
LAAO	5.60 × 10^4^	6.9	0.123	4.4	0.079	8.1	0.145	4.8	0.086	3.9	0.070	5.3	0.095	3.8	0.068
CTL	2.80 × 10^4^	2.5	0.089	2.9	0.104	2.4	0.086	8.0	0.286						
CRISP	2.30 × 10^4^	0.6	0.026	1.2	0.052	4.2	0.183	0.4	0.017	5.00	0.217	0.1	0.004	2.8	0.122
SVMPi	444.4	12.8	28.803	2.6	5.851	8.39	18.879	6.5	14.626	6.16	13.861	7.77	17.484	8.25	18.564

a The [high K49-PLA2/low PIII-SVMP] venom compositional dichotomy is highlighted in boldface.

The venom compositional profiles fit a linear correlation between the high abundance of K49-PLA_2_ and low concentration of P-III SVMP (adjusted *R*-squared of 0.4244; *p*-value: 8.136 × 10^–10^). A multiple linear model including the species in the correlation analysis confirmed the inverse proportional trend between high abundance of K49-PLA_2_ and low abundance of P-III SVMP (adjusted *R*-squared: 0.7423; *p*-value: 2.853 × 10^–14^).

Although the observed variation in venom composition is widely documented and has been attributed to several ecological drivers, including diet, no correlation was found between the venom compositional profiles and dietary habits. Thus, except for the nominal species *B. neuwiedi*, which has been described as a mammal specialist, all the other species within the *B. neuwiedi* complex exhibit a generalist diet comprising small mammals, anurans, lizards, snakes, birds, and centipedes.^91–95^

### 3.3 Functional Analysis

In line with their highly conserved proteomic profiles, snakes from the *B. neuwiedi* clade produce venoms with overlapping functional activities, which potentially explain the local (rapid edema formation, pain, inflammation, ecchymosis, hemorrhage, local myonecrosis, dermonecrosis, and blistering) and systemic effects (blood clotting perturbations, hypotensive shock and, in severe cases, kidney failure)^96–98^ observed in bothropic envenomations, including *B. diporus*,^20^ *B. neuwiedi*,^99,100^ *B. pauloensis*,^101^ and *B. mattogrossensis*.^38^ Panels A and B of Table 2 summarize the mean (±SD) or median (95% confidence intervals) values of the *in vitro* and *in vivo* biological activities, respectively, characterized in the pooled *B. neuwiedi* clade venoms sampled in this study. Values of the individual venoms comprising the species pools and statistical analysis of the functional assays are available in Supporting Information, S5.

**Table 2:** Summary of the Mean (± SD) or Median (95% Confidence Intervals) Values of the Biological Activities Characterized in *B. neuwiedi* Clade Venom Pools

	*B. mattogrossensis*	*B. pauloensis*	*B. pubescens*	*B. diporus*	*B. neuwiedi*	*B. marmoratus*	*B. erythromelas*
(A) *In vitro* venom activities							
phospholipase A_2_ (nmol/min/mg)	24.13 ± 4.03	55.84 ± 6.87	31.65 ± 3.90	39.01 ± 0.87	27.08 ± 5.70	26.52 ± 1.53	60.59 ± 9.193
collagenolytic activity (MCD, U/min/mg)	27.29 ± 0.54	6.19 ± 0.76	4.09 ± 0.54	4.30 ± 1.11	26.90 ± 2.29	54.86 ± 3.93	41.01 ± 4.50
thrombin-like activity (*A*_405 nm_)	1.36 ± 0.28	0.95 ± 0.01	1.10 ± 0.03	1.30 ± 0.02	1.06 ± 0.05	1.35 ± 0.05	0.24 ± 0.003
(B) *In vivo* venom activities							
hemorrhagic (MHD, μg/18–22g mouse)	2.99 ± 0.27	2.96 ± 0.47	4.69 ± 0.56	6.48 ± 0.05	2.89 ± 0.32	3.85 ± 0.30	1.13 ± 0.35
edematogenic activity (D paw thickness)	126.1 (99.86–152.4)	174.5 (154.3–194.6)	87.35 (65.36–109.3)	125.7 (133.9–176.7)	155.3 (133.9–176.7)	142.4 (110.3–174.5)	71.38 (45.56–97.20)
myotoxicity (creatine kinase, U/L)	511.51 ± 40.49	365.82 ± 189.04	1491.99 ± 262.45	458.24 ± 50.67	299.79 ± 62.47	408.12 ± 42.75	145.86 ± 43.51
lethal (i.p. LD_50_, μgV/g mouse body weight)	3.29 (2.55–4.46)	2.38 (1.45–3.66)	2.47 (1.49–3.88)	2.01 (1.35–2.68)	2.47 (1.72–3.62)	2.91 (1.84–4.51)	4.87 (3.34–6.64)
(C) Antivenomics							
mg venom bound/g AV	93.08	n.a.^a^	75.87	n.d.^b^	165.67	102.9	n.d.^c^
mg venom bound/mL AV	4.32		3.52		7.70	4.78	
(D) *In vivo* antivenom neutralization							
ED_50_ (mgV/g SAB)	143.4 (115.7–188.6)	135.9 (111.3–174.8)	129.5 (86.9–180.9)	281.4 (228.8–365.2)	210.4 (171.4–272.4)	154.5 (114.6–237.0)	161.8 (111.4–295.8)
potency [P] (mgV/g SAB AV)	114.7 (92.5–150.9)	108.8 (89.0–139.9)	103.6 (69.5–144.7)	225.1 (183.1–292.1)	168.3 (137.1–217.9)	123.6 (91.7–189.6)	129.4 (89.1–236.7)
% antitoxin neutralizing antibodies	123.23		136.55		101.59	120.12	
(E) Venom yield and SAB vials/bite							
venom yield (mg)	88.5	90.5	36.5	134.0 ± 68	61.3	43.8	36.3
vials SAB/venom yield	1.7	1.8	0.8	1.3	0.8	0.8	0.6
g F(ab’)_2_/vial SAB antivenom	0.4645						

a na., data not available.

b Not determined in this study; antivenomics assessment in Gay et al., Toxins (Basel). 2016, Dec 25; 8(1):9. doi: 10.3390/toxins8010009.

c Not determined in this study; antivenomics assessment in Jorge et al., J. Proteomics. 2015 Jan 30; 114:93–114. doi: 10.1016/j.jprot.2014.11.011.

Local damage is mainly caused by extracellular matrix-degrading Zn^2+^-dependent PIII-SVMP^102^ and cytolytic PLA_2_ molecules.^103^ Myonecrosis, edema, inflammation, and acute muscle damage are also widely correlated with PLA_2_ molecules.^104,105^ Thus, acidic D49-PLA2s AFJ79207 and AFJ79208 isolated from *B. diporus* venom exhibited edema-inducing activity but lacked myotoxicity,^106^ whereas basic myotoxic K49-PLA_2_ myotoxin I from *B. diporus* collected in the provinces of Santiago del Estero, Corrientes, and Misiones (Argentina) showed myotoxic, cytolytic, and edema-inducing activities.^107^ Thrombin-like serine proteinases targeting coagulation factors, alpha-fibrino­(geno)­lytic PI-SVMPs, platelet aggregation inhibitory disintegrins, and CTL molecules synergistically potentiate the activity of hemorrhagic PIII-SVMPs, resulting in increased incidence of systemic bleeding, and interact with components of the hemostatic system, contributing to blood clotting perturbations.^108–113^ Snake venom VEGF-mediated hypotension and venom spread through an increase in vascular permeability may act synergistically contributing to toxin dispersion, enhancement of the hemotoxicity of the venom.^114,115^ In addition, BPPs are inhibitors of the angiotensin I-converting enzyme, resulting in a net increase of the hypotensive effect of the circulating bradykinin, thereby contributing to cardiovascular shock in the snake’s prey or human victim.^116–118^

#### 3.3.1 Collagenolytic Activity and MHD

Our present comparative study of biological activities across *B. neuwiedi* clade venoms revealed large individual variability of collagenolytic activity, particularly in the venom of *B. neuwiedi* (ranging from 1.807 to 89.422 U/mg/min) (Figure 5A; Supporting Information, S5). Our results showed a correlation (*R* ^2^ = 0.8546 and *p*-value = 0.0029) between low collagenolytic activity in the venoms of *B. pauloensis*, *B. pubescens*, and *B. diporus* (Figure 5A) and the absence of a PIII-SVMP peak eluting at 83 min in the RP-HPLC profiles of the venoms exhibiting high collagenolytic activity—*B. mattogrossensis* (Figure 2), *B. neuwiedi*, *B. marmoratus,* and *B. erythromelas* (Figure 4). Interestingly, *B. pauloensis*, *B. pubescens*, and *B. diporus* form a sister subclade of *B. mattogrossensis* in the phylogenetic relationship of the *B. neuwiedi* complex (Figure 1A and B), suggesting that the PIII-SVMP/collagenolytic activity correlation may have emerged at the base of the monophyletic [*B. mattogrossensis*, *B. pauloensis*, *B. pubescens*, and *B. diporus*] lineage. Identical PIII-SVMP has been associated with the collagenolytic activity of *B. jararaca* ^119^ and *B. moojeni* ^47,120^ venoms, further pointing to this PIII-SVMP [83 min] as a collagenolytic metalloprotease. In concordance with this hypothesis, a statistically significant inverse correlation (Pearson *r* = −0.7554; *R* square = 0.5706, *p*-value = 0.0496) was established between the venoms’ collagenolytic activities (Figure 5A) and their MHD (Figure 5B), which results from disruption of the integrity of the microvasculature by hemorrhagic PIII-SVMPs.^119,121^

**Figure 5:**
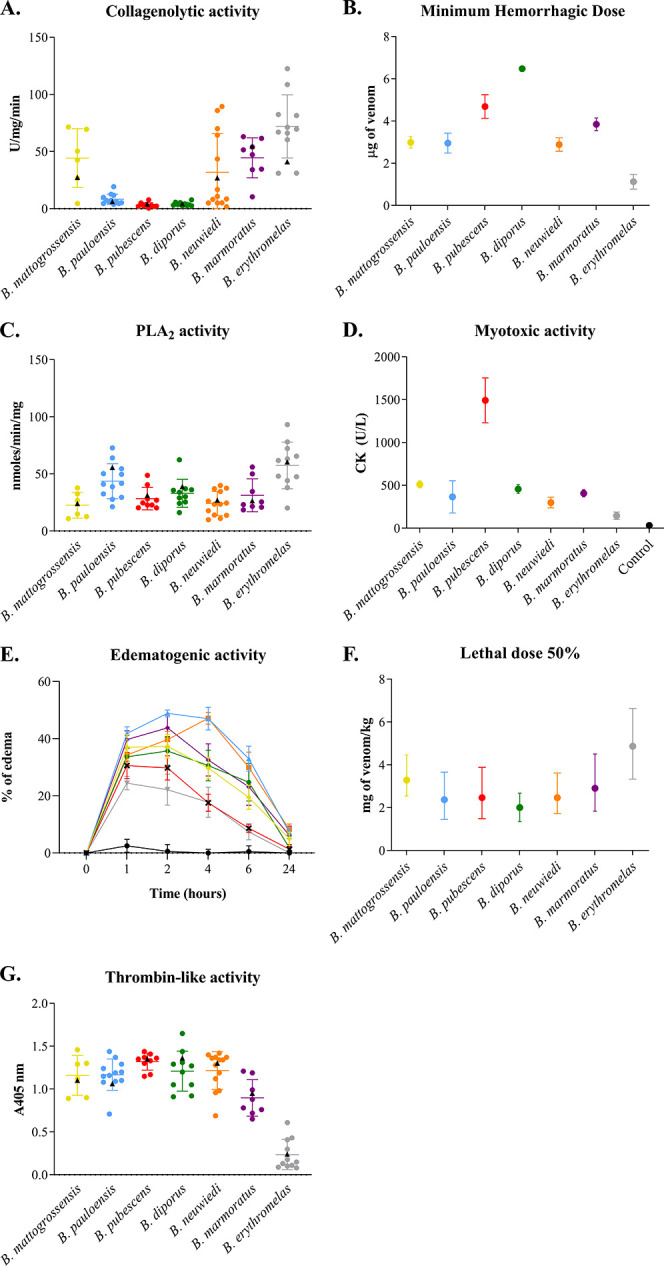
Enzymatic and *in vivo* activities of individual and pooled venoms from *B. neuwiedi* clade. (A) Collagenolytic activity. (B) Minimum Hemorrhagic Dose (MHD). (C) PLA_2_ activity. (D) Myotoxic activity, represented by CK liberation after 3 h of i.m. venom injection. (E) Edematogenic activity, expressed by % of edema induced by snake venom after 1, 2, 4, 6, and 24 h of injection. (F) Lethal dose 50% (LD_50_), with superior and inferior limits expressed by error bars. (G) Thrombin-like activity using S-2238 substrate (Chromogenix). Methodological procedures are described in Experimental Section.

#### 3.3.2 PLA_2_ Hydrolytic and Myotoxic Activities, Edema Formation, and Tissue Damage

In line with its highest content of catalytically active D49-PLA_2_, and lack of K49-PLA_2_ (Figure 4, Table 1) and myotoxic activity,^122,123^ *B. erythromelas* venom had the highest phospholipasic activity on the chromogenic substrate NOBA (Table 2, Figure 5C). Myotoxic activity was determined by quantification of serum CK after venom injection and, in concordance with previous studies,^122,123^ *B. erythromelas* and *B. neuwiedi* venoms yielded values not statistically different from saline (negative control) (Table 2 and Figure 5D). In contrast, *B. pubescens* venom showed the greatest myotoxic activity (Table 2 and Figure 5D), in accordance with the high content of K49-PLA_2_ (Figure 4, Table 1). However, synergistically acting D49- and K49-PLA_2_ molecules have been characterized in *B. mattogrossensis* ^124^ and *B. diporus* ^125,126^ venoms, where the hydrolytic activity of D49-PLA_2_s may destabilize the cell membrane integrity, thereby facilitating the myotoxic activity of K49-PLA_2_ and increasing venom toxicity.^125,127^

Local tissue damage commonly observed in bothropic envenomings is caused by the myotoxic, hemorrhagic, and edematogenic action of the venom components.^2^ Histopathological analysis of the gastrocnemius muscle (Figure 6) revealed that all the *B. neuwiedi* clade venoms triggered an inflammatory edematogenic response with massive leukocyte infiltration, hemorrhage, and tissue damage described in bothropic envenomings.^128–130^ *B. erythromelas* venom produced intense hemorrhage seemingly due to its high abundance of hemorrhagic toxins.^131^ Gastrocnemius muscle injected with *B. mattogrossensis*, *B. pauloensis,* or *B. pubescens* venom presented necrotic muscular fibers, evidenced by the presence of pyknotic nuclei (Figure 6, panels B–D).

**Figure 6:**
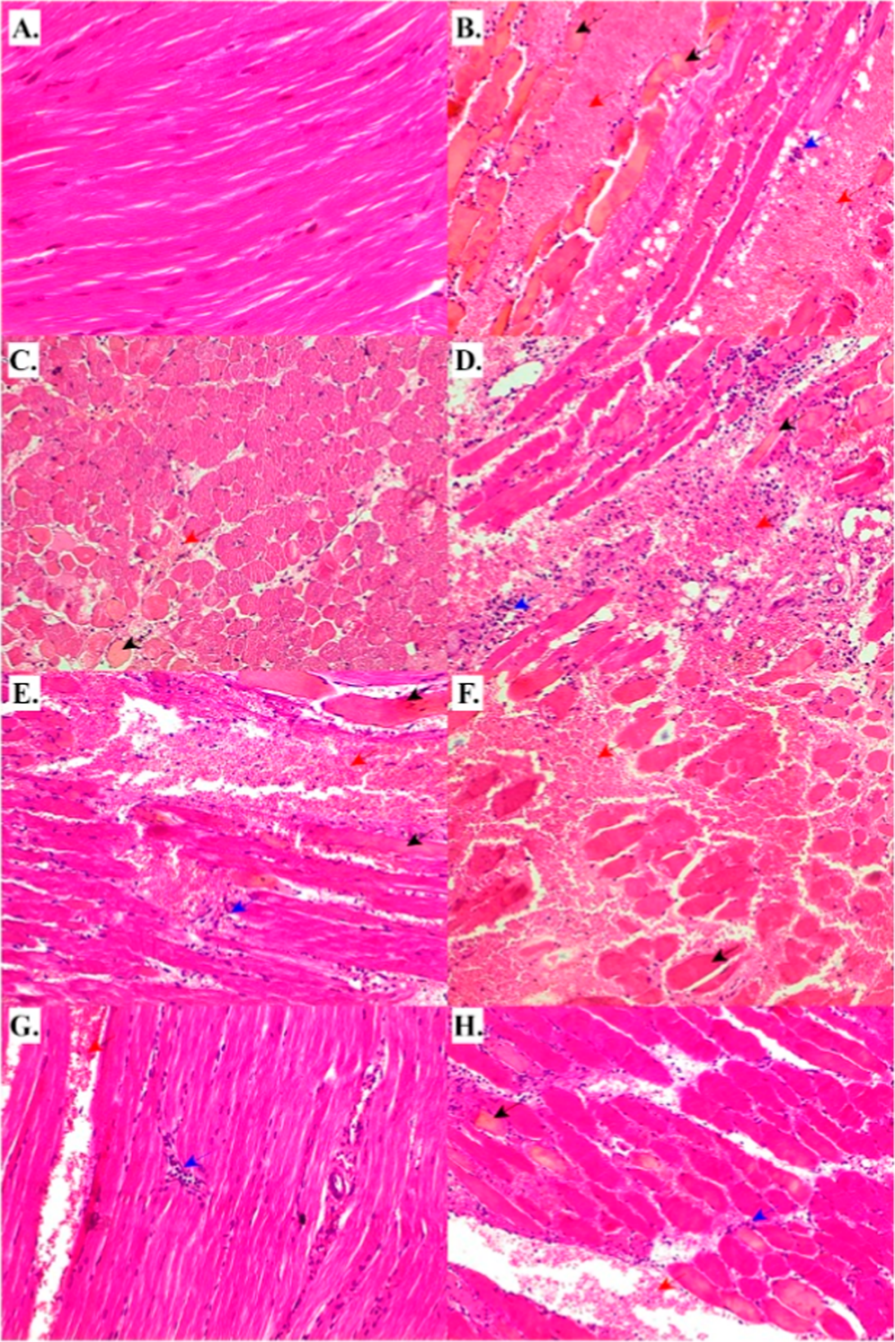
Hystopathology of gastrocnemius muscle of mice injected with venom from species of *B. neuwiedi* clade. (A) Control group injected with 0.85% sterile saline solution (40×). (B) *B. mattogrossensis* venom (20×). (C) *B. pauloensis* venom (20×). (D) *B. pubescens* venom (20×). (E) *B. diporus* venom (20×). (F) *B. neuwiedi* venom (20×). (G) *B. marmoratus* venom (20×) and (H) *B. erythromelas* venom (20×). Black arrows indicate areas of necrosis; red arrows show hemorrhagic regions; and blue arrows show inflammatory infiltrate. Edema can be seen in all tissues analyzed.

Among the seven species studied, *B. pauloensis* produces the most edematogenic venom, while *B. erythromelas* has the least. The peak of edema formation was reached after 2 h of venom injection for all the species, except *B. neuwiedi*, which happened after 4 h, and *B. erythromelas*, which reached the peak after just 1 h. After 6 h of venom application, *B. erythromelas* and *B. pubescens* no longer differ from saline, indicating complete edema remission. Conversely, complete edema resolution occurred after 24 h in *B. pauloensis*, *B. neuwiedi*, *B. mattogrossensis*, *B. marmoratus,* and *B. diporus* (Figure 5E). This temporal edematogenic profile is similar to reports from other *Bothrops* species.^25,61,119,132,133^ The statistical analysis of the time-course edematogenic effect is available in Supporting Information, S5.

#### 3.3.3 Thrombin-Like Activity

Hemostatic disorders caused by snake venom thrombin-like SVSP enzymes play a key role in the pathophysiology of envenomation and prey capture.^134^ Snake venom thrombin-like enzymes clot fibrinogen, an essential protein for fibrin net formation and platelet aggregation,^135^ thereby contributing to the depletion of circulating clottable fibrinogen, causing venom-induced consumption coagulopathy and hemorrhagic syndrome, typical features of *Bothrops* sp. snake envenoming.^136^ Except for *B. erythromelas*, which presented the lowest venom thrombin-like activity (Figure 5G; Table 2), there is no difference in thrombin-like activity among the *B. neuwiedi* clade species studied (for *p*-values comparison, access Supporting Information, S5). The low thrombin-like activity of *B. erythromelas* venom was expected from the low relative abundance (7% of the venom proteome) of SVSP.^24^ In addition, Nahas et al. (1979)^137^ and Furtado et al. (1991)^138^ demonstrated lack of thrombin-like activity in *B. erythromelas* venom, and Lotto et al. (2021)^135^ described the deletion of the snake venom thrombin-like gene in *B. erythromelas*. Another factor that may contribute to the lack of thrombin-like activity is the high fibrinogenolytic activity of *B. erythromelas* venom that may counteract the thrombin-like activity.^131^

#### 3.3.4 Venom Lethal Activity across the *B. neuwiedi* Species Clade

Intravenous murine Lethal Dose 50% (LD_50_) values for the venom of the species of the *B. neuwiedi* clade ranged between 2.01 (CI95%: 1.35–2.68) mg/kg (*B. diporus*) and 4.87 (CI95%: 3.34–6.64) mg/kg (*B. erythromelas*) (Table 2B). These figures are comparable to LD_50_s reported for other *Bothrops* species.^51,119,120,139^

Hemorrhagic activity has been pointed to play a central role in physiopathology of *B. asper* ^140^ and *Bothrops atrox* ^51^ snakebites, and the hemorrhagic activity of nine *Bothrops* species has been positively correlated with the venoms’ lethality by Ferreira et al. (1992) with a *R* of 0.54.^141^ However, our results show an inverse correlation between hemorrhagic activity and lethality (Pearson *r* = −0.7799; *R* ^2^ = 0.6082 and *p*-value = 0.0386) for the venoms of the *B. neuwiedi* clade (Figure 7A). Notwithstanding the low sample size, such trend reminds the functional dichotomy described in rattlesnakes, where type I venoms with high hemorrhagic activity were less toxic, while highly toxic (type II) venoms showed low protease activity and higher neurotoxic or myotoxic activities.^142,143^ The LD_50_s from species in the *B. neuwiedi* clade also show inverse correlation (Pearson *r* = −0.3665; *R* ^2^ = 0.1344, *p*-value = 0.4187) with their venom myotoxicity (Figure 7B). Clearly, the role of synergistic pathophysiological effects caused in bothropic envenomings,^2,119^ i.e., coagulopathy, deserves further investigation.

**Figure 7:**
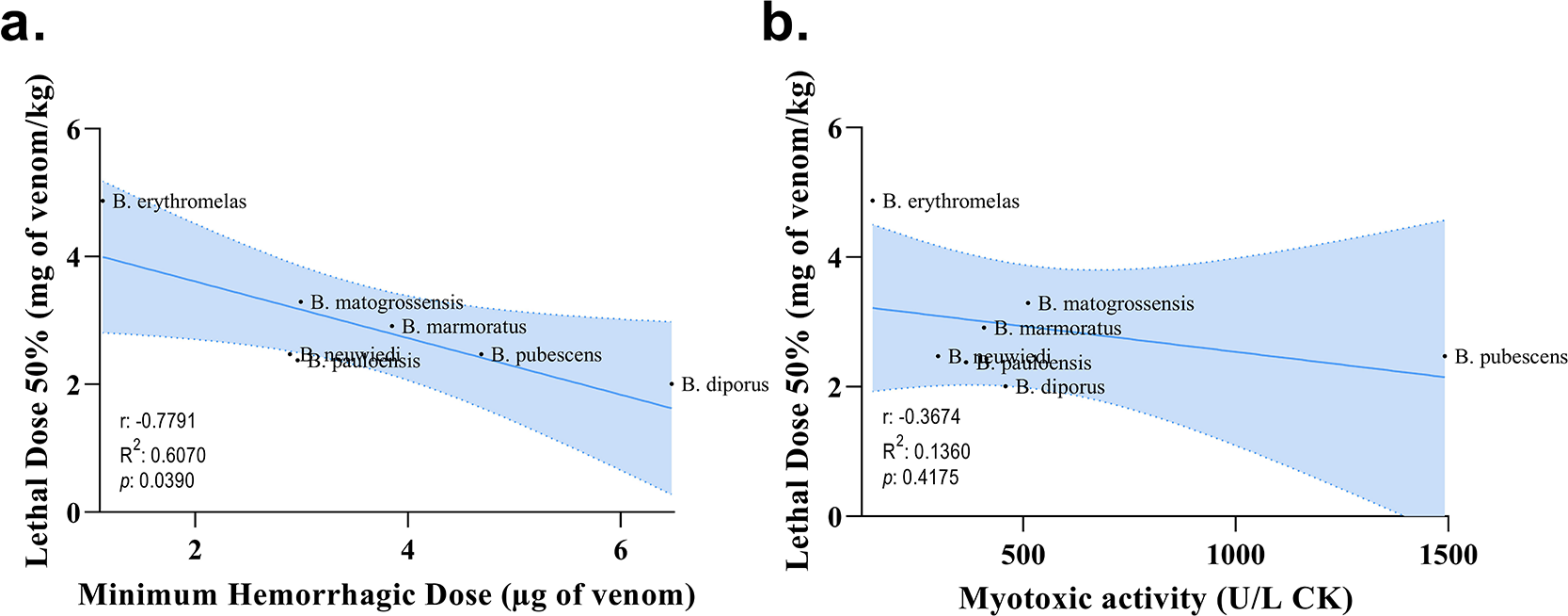
Correlation analysis between Lethal Dose 50% and Minimum Hemorrhagic Dose and myotoxicity. Pearson correlation test was performed between (A) LD_50_ and MHD and (B) LD_50_ and myotoxicity.

### 3.4 Antivenomics Assessment of the Paraspecific Immunorecognition and Lethality Neutralization Potency of the Brazilian Pentabothropic Antivenom toward Venoms from the *B. neuwiedi* Clade Species

The ability of SAB pentabothropic antivenom to recognize a broad spectrum of medically important bothropic venoms, including those of *B. diporus* ^19^ and *B. erythromelas* ^24^ within the *B. neuwiedi* clade, has been documented in works spanning the last three decades.^36,38,40,144–147^ Here, we have applied third-generation antivenomics (3GA) to assess the toxin-resolved paraspecific immunoreactivity of the SAB antivenom toward the venoms of *B. mattogrossensis*, *B. neuwiedi*, *B. pubescens*, and *B. marmoratus* (Figure 8).

**Figure 8:**
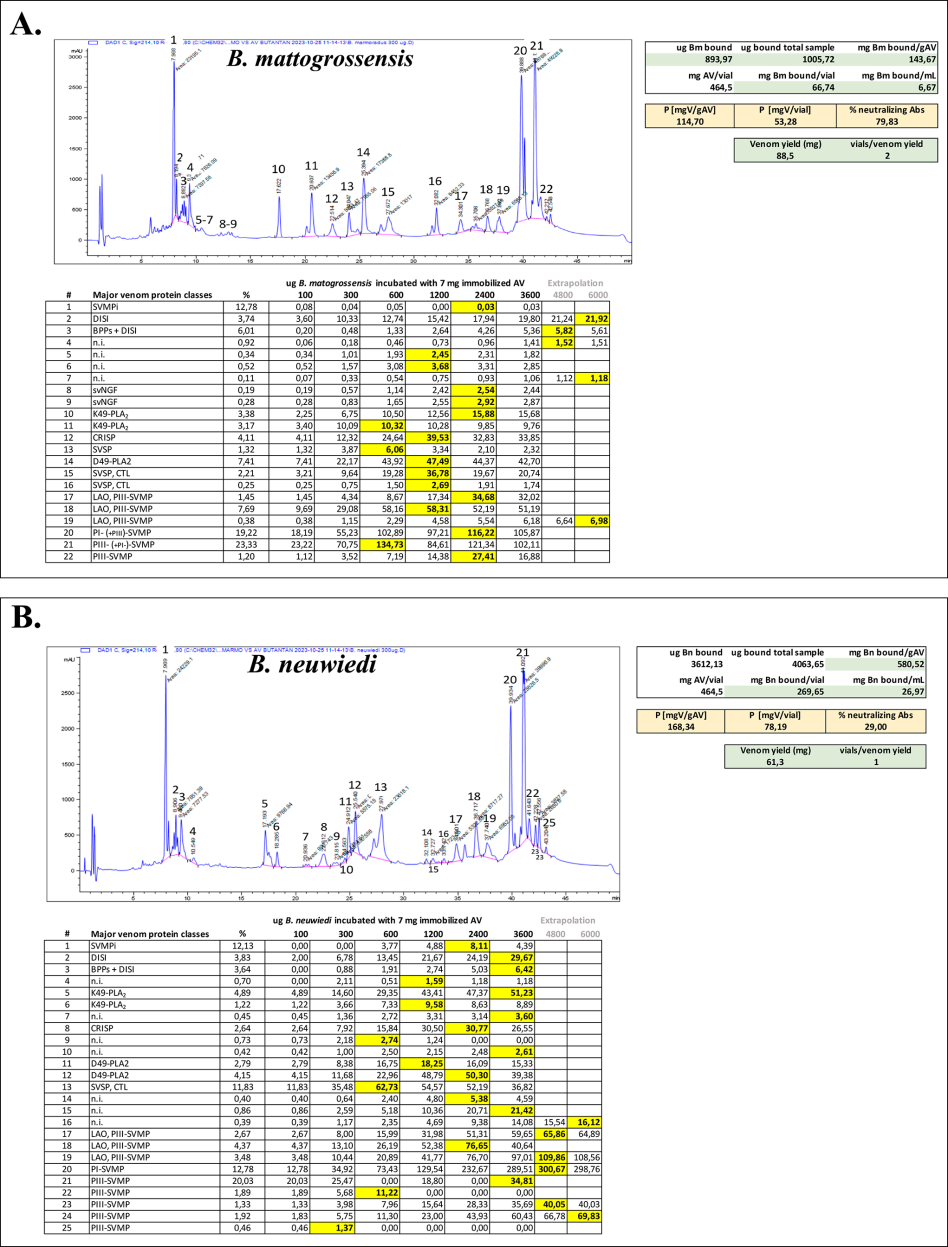

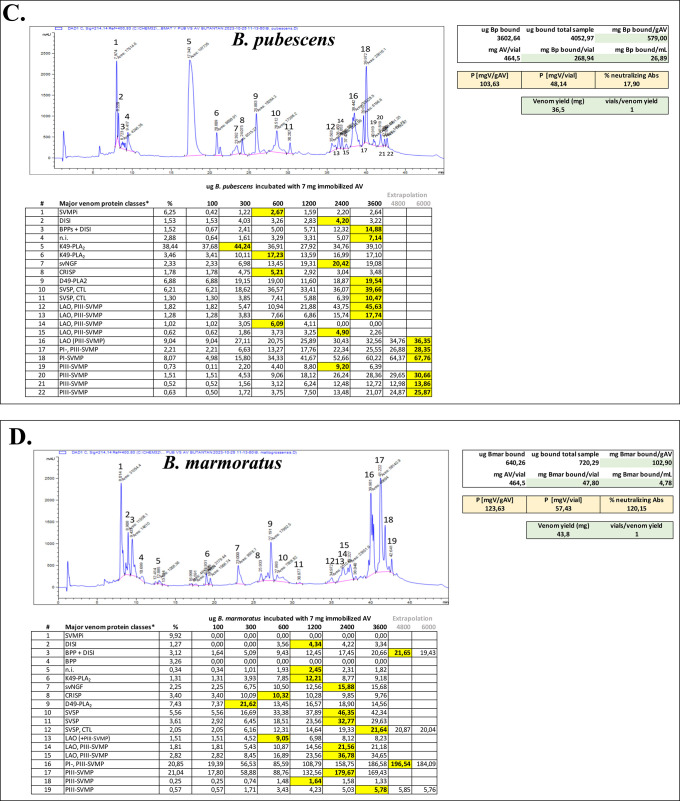
Antivenomics of Brazilian bothropic polyvalent antivenom from Instituto Butantan against venoms of the *Bothrops neuwiedi* clade. Concentration-dependent and toxin-resolved maximal venom immunoretention for each RP-HPLC fraction in the affinity matrix is highlighted in boldface and yellow background. Binding saturation was computed by extrapolation from data modeled in Excel to degree 2 polynomial functions. Antivenom’s binding capacity (in mg venom (V)/g antivenom (AV), mgV/vial, and mgV/mL AV) is displayed in the boxes right to the chromatogram. Acronyms: SVMPi, tripeptide inhibitors of snake venom metalloproteinases (SVMP); DISI, disintegrin; BPP, bradykinin potentiating peptide; PLA_2_, phospholipase A_2_; svNGF, snake venom nerve growth factor; svVEGF, snake venom vascular endothelial growth factor; CRISP, cysteine-rich secretory protein; SVSP, snake venom serine proteinase; CTL, C-type lectin-like; LAO, l-amino acid oxidase; PDE, phosphodiesterase; 5NT, 5′-nucleotidase; and PIII- and PI-SVMP, SVMPs of class PI- and PIII, respectively.

The antivenomic analyses, displayed in Figure 8 and Supporting Information, S7–S10, showed extensive cross-reactivity across all of the toxin-resolved venom proteomes. Table 2C provides a summary of the maximum binding capacities (in mg venom/g antivenom and mg V/mL AV) of the Brazilian SAB antivenom toward *B. mattogrossensis*, *B. neuwiedi*, *B. pubescens*, and *B. marmoratus* venoms. The antivenom’s binding capacity toward *B. neuwiedi* venoms was comparable to its ability to immunocapture the venom of the reference venom (173.1 mg *B. jararaca* venom/g SAB antivenom): 165.7 mg (*B. neuwiedi*), but was significantly lower for the other venoms tested: 102.9 mg (*B. marmoratus*), 93.1 mg (*B. mattogrossensis*), and 75.9 mg (*B. pubescens*) of venom/g SAB. However, antivenom binding capacity is not necessarily a proxy for neutralization potency, and thus translating in vitro data to a clinical scenario is not straightforward. Therefore, the effective median dose (ED_50_) of the SAB antivenom toward 5 LD_50_s of each of the seven *B. neuwiedi* clade venoms was determined in the murine model (Table 2D; Supporting Information, S6). The ratio between the venom neutralizing potency [P] and the maximal toxin-binding capacity (MaxBind) of an antivenom represents the proportion of toxin-binding antibodies that contribute to its neutralization potency. For all the venoms analyzed, this figure was >100%: *B. mattogrossensis* (123.6%), *B. pubescens* (136.6%), *B. neuwiedi* (101.6%), and *B. marmoratus* (120.1%). This apparent counterintuitive result is explained by considering that the different venom proteins may contribute asymmetrically to the toxicovenomic profile of the venom: if neutralizing a subset of venom toxins to below their lethality threshold the antivenom abrogates the lethal potential of the whole venom, *P* = [MaxBind/(relative abundance (%) of the neutralized toxins)], and thus the mathematical result of (*P*/MaxBind) × 100 > 100%.

Averaged venom yields for the venoms of the *B. neuwiedi* group gathered from historical archives of the Laboratório de Herpetologia, Instituto Butantan, São Paulo, Brazil, and Instituto Nacional de Producción de Biológicos, A.N.L.I.S. “Dr. Carlos G. Malbrán”, Buenos Aires, Argentina, are *B. mattogrossensis* (88.5 mg), *B. pauloensis* (90.5 mg), *B. pubescens* (36.5 mg), *B. diporus* (134 ± 68 mg), *B. neuwiedi* (61.3 mg), *B. marmoratus* (43.8 mg), and *B. erythromelas* (36.3 mg) (Table 2E and Supporting Information, S6). These figures suggest that treatment of bites by species of the *B. neuwiedi* group deploying an average amount of venom would require 1–2 vials of SAB antivenom (Table 2E and Supporting Information, S6). Such remarkable paraspecificity is in line with previous reports showing neutralization of *B. erythromelas* venom activities,^148^ hemorrhage, edema, and lethal activity by SAB antivenom.^149^

### 3.5 Concluding Remarks

This work presents the first comparative characterization of the venoms of seven species from the *B. neuwiedi* clade after its taxonomic revision. Structural and functional venom profiling revealed intraspecific and interspecific variability in the context of the highly conserved qualitative *Bothrops* venom pattern. Of note, all the *B. neuwiedi* clade venoms analyzed showed a higher concentration of PI-SVMP than PIII-SVMPs. Also, our work unveiled a [high K49-PLA_2_/low PIII-SVMP] venom compositional dichotomy in the monophyletic subclade (*B. mattogrossensis*, (*B. pauloensis*, (*B. pubescens* and *B. diporus*))) of the *B. neuwiedi* clade. However, the evolutionary origin and ecological consequences of this venom variability deserve further research. In the Carrasco et al. (2023) phylogeny,^18^ the *B. jararaca* group (*B. sazimai* ((*B. alcatraz* and *B. otavoi*) (*B. insularis* and *B. jararaca*))) is the sister clade of the *B. neuwiedi* group. The venoms of *B. insularis* ^150^ and *B. alcatraz* ^151^ contain exclusively acidic D49-PLA_2_; *B. jararaca* venom expresses both D49-PLA_2_ and basic K49-PLA_2_ molecules;^29^ and the venoms’ proteomes of *B. sazimai* and *B. otavoi* have not been characterized.

The remarkable neutralization capacity exhibited by the Brazilian SAB antivenom against the lethality of all the *B. neuwiedi* clade venoms sampled is in line with previous findings showing broad paraspecific neutralization of different bothropic antivenoms toward *Bothrops* envenomings across South American countries.^32–42^ While the medical efficacy of SAB already suggested broad cross-reactivity between venoms of the genus *Bothrops*, our current antivenomics results highlight the immunological basis underlying the antivenom’s paraspecific activity, namely, the conservation across the 12–16 million years of natural history of genus *Bothrops* ^90,146^ of immunoreactive epitopes on the major venom toxin families. Specifically, significant cross-reactivity was observed for the major toxin classes: K49-PLA2 (52.5 ± 24.4%), D49-PLA2 (52.7 ± 31.5%), SVSP (58.5 ± 30.0%), PIII-SVMP (49.5 ± 20.6%), and PI-SVMP (40.1 ± 34.2%). Our antivenomics data allow the rationalization, in molecular terms, of the conclusions of *in vivo* neutralization assays of the ability of SAB pentabothropic antivenom to recognize a broad spectrum of medically important bothropic venoms.^36,38,40,144–147^ This, in turn, provides clues for improving the potency and expanding the spectrum of the clinical applicability of the Brazilian antibothropic polyvalent SAB antivenom.^152^

## Supplementary Material



## Data Availability

The mass spectrometry proteomics data have been deposited to the ProteomeXchange Consortium^58^ via the PRIDE^59^ partner repository with the data set identifier PXD066646.

## Notes

Gender, geographical origin, and morphological characteristics of the individual *B. neuwiedi* clade snakes; snake venomics analysis of *B. matogrossensis* venom; RP HPLC profiles of the venoms gathered from individual snakes from the *B. neuwiedi* clade; comparative RP HPLC profiles of the *B. neuwiedi* clade’ snake venoms; statistical analysis of *in vitro* and *in vivo* assays of venom biological activities; venoms’ LD50 and ED50 of the Brazilian Instituto Butantan’ soro antibothrópico pentavalente against venoms of the *B. neuwiedi* clade; antivenomics analysis of the Brazilian pentabothropic antivenom against the venom of *B. matogrossensis*; antivenomics analysis of the Brazilian pentabothropic antivenom against the venom of *B. neuwiedi*; antivenomics analysis of the Brazilian pentabothropic antivenom against the venom of *B. pubescens*; and antivenomics analysis of the Brazilian pentabothropic antivenom against the venom of *B. marmoratus* (XLSX)

## References

[1] WHO . World Health Organization. http://www.who.int/snakebites/disease/en/. (accessed 03 21, 2025).

[2] Gutiérrez J. M., Calvete J. J., Habib A. G., Harrison R. A., Williams D. J., Warrell D. A. (2017). Snakebite
Envenoming. Nat. Rev.
Dis Primers.

[3] Harrison R. A., Hargreaves A., Wagstaff S. C., Faragher B., Lalloo D. G. (2009). Snake Envenoming:
A Disease of Poverty. PLoS Neglected Trop. Dis..

[4] Potet J., Beran D., Ray N., Alcoba G., Habib A. G., Iliyasu G., Waldmann B., Ralph R., Faiz M. A., Monteiro W. M., de Almeida
Gonçalves Sachett J., di Fabio J. L., Cortés M. d. l.
Á., Brown N. I., Williams D. J. (2021). Access to Antivenoms in the Developing World: A Multidisciplinary
Analysis. Toxicon:X.

[5] Williams D. J., Faiz M. A., Abela-Ridder B., Ainsworth S., Bulfone T. C., Nickerson A. D., Habib A. G., Junghanss T., Fan H. W., Turner M., Harrison R. A., Warrell D. A. (2019). Strategy
for a Globally Coordinated Response to a Priority Neglected Tropical
Disease: Snakebite Envenoming. PLoS Neglected
Trop. Dis..

[6] Kalil, J. ; Fan, H. W. Production and Utilization of Snake Antivenoms in South America. In Toxins and Drug Discovery; Cruz, L. J. , Luo, S. , Eds., 2017; pp 81–100.

[7] Uetz, P. ; Freed, P. ; Aguilar, R. ; Reyes, F. ; Kudera, J. ; Hošek, J. The Reptile Database. http://www.reptile-database.org (accessed 03 26, 2025).

[8] Campbell, J. A. ; Lamar, W. W. The Venomous Reptiles of Latin America; Comstock Publishing Associates: Ithaca, NY, 1989.

[9] da
Silva W. R. G. B., de Siqueira Santos L., Lira D., de Oliveira
Luna K. P., Fook S. M. L., Alves R. R. N. (2023). Who Are the Most
Affected by *Bothrops* Snakebite Envenoming in Brazil?
A Clinical-Epidemiological Profile Study among the Regions of the
Country. PLoS Neglected Trop. Dis..

[10] da Saúde, M.´rio. Sistema de Informação de Agravos de Notificação. Acidente Por Animais Peçonhentos, Serpentes Peçonhentas. Notificações Registradas: Banco de Dados, 2023. http://tabnet.datasus.gov.br/cgi/deftohtm.exe?sinannet/cnv/animaisbr.def.

[11] Carrasco P. A., Grazziotin F. G., Farfán R. S. C., Koch C., Antonio
Ochoa J., Scrocchi G. J., Leynaud G. C., Chaparro J. C. (2019). A New Species
of *Bothrops* (Serpentes: Viperidae: Crotalinae) from
Pampas Del Heath, Southeastern Peru, with Comments on the Systematics
of the *Bothrops neuwiedi* Species Group. Zootaxa.

[12] Melgarejo, A. R. Serpentes Peçonhentas Do Brasil. In Animais peçonhentos no Brasil; Cardoso, J. L. C. , França, F. O. S. , Wen, F. H. , Málaque, C. M. S. , Haddad, Jr. V. , Eds.; São Paulo, 2009; pp 42–70.Savier

[13] Spix, J. B. von. ; Wagler, J. G. Serpentum Brasiliensium Species Novae Ou. Histoire Naturelle Des Espèces Nouvelles de Serpens: Recueillies et Observées Pendant Le Voyage Dans l’intérieur Du Brésil Dans Les Années 1817, 1818, 1820, 1824.

[14] Campbell, J. A. ; Lamar, W. The Venomous Reptiles of the Western Hemisphere; Commstock (Cornell University Press: Ithaca NY, 2004.

[15] Machado T., Silva V. X., Silva M. J. de J. (2014). Phylogenetic
Relationships within *Bothrops neuwiedi* Group (Serpentes,
Squamata): Geographically
Highly-Structured Lineages, Evidence of Introgressive Hybridization
and Neogene/Quaternary Diversification. Mol.
Phylogenet. Evol..

[16] Silva V. X. d., Rodrigues M. T. (2008). Taxonomic
Revision of the *Bothrops neuwiedi* Complex (Serpentes,
Viperidae) with Description of a New Species. Phyllomedusa.

[17] Alencar L. R. V., Quental T. B., Grazziotin F. G., Alfaro M. L., Martins M., Venzon M., Zaher H. (2016). Diversification
in Vipers: Phylogenetic
Relationships, Time of Divergence and Shifts in Speciation Rates. Mol. Phylogenet. Evol..

[18] Carrasco P. A., Koch C., Grazziotin F. G., Venegas P. J., Chaparro J. C., Scrocchi G. J., Salazar-Valenzuela D., Leynaud G. C., Mattoni C. I. (2023). Total-evidence
Phylogeny and Evolutionary Morphology of New World Pitvipers (Serpentes:
Viperidae: Crotalinae). Cladistics.

[19] Gay C., Sanz L., Calvete J. J., Pla D. (2015). Snake Venomics and
Antivenomics of *Bothrops diporus*, a Medically Important
Pitviper in Northeastern Argentina. Toxins.

[20] de
Oliveira V. C., Lanari L. C., Hajos S. E., De Roodt A. R. (2011). Toxicity
of *Bothrops neuwiedi* Complex (“Yarará
Chica”) Venom from Different Regions of Argentina (Serpentes,
Viperidae). Toxicon.

[21] Rodrigues R. S., Boldrini-França J., Fonseca F. P. P., de
la Torre P., Henrique-Silva F., Sanz L., Calvete J. J., Rodrigues V. M. (2012). Combined Snake Venomics and Venom Gland Transcriptomic
Analysis of *Bothropoides pauloensis*. J. Proteomics.

[22] Tasima L. J., Hatakeyama D. M., Aguiar W. da S., Lima E. O. V. de, Miyamoto J. G., Tashima A. K., Sant’Anna S. S., Grego K. F., Morais-Zani K. de, Tanaka-Azevedo A. M. (2022). Analyzing
the Influence of Age and Sex in *Bothrops pauloensis* Snake Venom. Toxicon.

[23] Rangel D. L., Melani R. D., Carvalho E. L., Boldo J. T., Gomes
dos Santos T., Kelleher N. L., Pinto P. M. (2022). Venom Characterization
of the Brazilian Pampa Snake *Bothrops pubescens* by
Top-down and Bottom-up Proteomics. Toxicon.

[24] Jorge R. J. B., Monteiro H. S. A., Gonçalves-Machado L., Guarnieri M. C., Ximenes R. M., Borges-Nojosa D. M., Luna K. P. de O., Zingali R. B., Corrêa-Netto C., Gutiérrez J. M., Sanz L., Calvete J. J., Pla D. (2015). Venomics and
Antivenomics of *Bothrops erythromelas* from Five Geographic
Populations within the Caatinga Ecoregion of Northeastern Brazil. J. Proteomics.

[25] Rodrigues V. M., Soares A. M., Mancin A. C., Fontes M. R. M., Homsi-Brandeburgo M.
I., Giglio J. R. (1998). Geographic
Variations in the Composition of Myotoxins
from *Bothrops neuwiedi* Snake Venoms: Biochemical
Characterization and Biological Activity. Comp.
Biochem. Physiol., Part A:Mol. Integr. Physiol..

[26] Lopes D. S., Baldo C., de Freitas Oliveira C., Machado de Alcântara T., Dias Oliveira J. D., Gourlart L. R., Hamaguchi A., Homsi-Brandeburgo M. I., Moura-da-Silva A. M., Clissa P. B., de Melo
Rodrigues V. (2009). Characterization of Inflammatory Reaction Induced by
Neuwiedase, a P-I Metalloproteinase Isolated from *Bothrops
neuwiedi* Venom. Toxicon.

[27] Bernardoni J. L., Sousa L. F., Wermelinger L. S., Lopes A. S., Prezoto B. C., Serrano S. M. T., Zingali R. B., Moura-da-Silva A. M. (2014). Functional
Variability of Snake Venom Metalloproteinases: Adaptive Advantages
in Targeting Different Prey and Implications for Human Envenomation. PLoS One.

[28] Instituto Butantan. Website. http://www.butantan.gov.br/. (accessed 03 19 2025).

[29] Gonçalves-Machado L., Pla D., Sanz L., Jorge R. J. B., Leitão-De-Araújo M., Alves M. L. M., Alvares D. J., De Miranda J., Nowatzki J., de Morais-Zani K., Fernandes W., Tanaka-Azevedo A. M., Fernández J., Zingali R. B., Gutiérrez J. M., Corrêa-Netto C., Calvete J. J. (2016). Combined Venomics, Venom Gland Transcriptomics,
Bioactivities, and Antivenomics of Two *Bothrops jararaca* Populations from Geographic Isolated Regions within the Brazilian
Atlantic Rainforest. J. Proteomics.

[30] Brasil, M. da S. Normas de Produção e Controle de Qualidade de Soros Antiofídicos: Brazil, 1996; Vol. 234, pp 491–512.

[31] Raw, I. ; Guidolin, R. ; Higashi, H. C. ; Kelen, E. M. A. Antivenins in Brazil: Prepartion. In Handbook of natural toxins; Routledge, 2018; pp 557–581.

[32] Moura-da-Silva A. M., D’Império-Lima M. R., Nishikawa A. K., Brodskyn C. I., Dos-Santos M. C., Furtado M. F. D., Dias-da-Silva W., Mota I. (1990). Antigenic Cross-Reactivity
of Venoms Obtained from Snakes of Genus *Bothrops*. Toxicon.

[33] De
Roodt A. R., Dolab J. A., Ferna T., Ndez A. ^., Segre L., Hajos S. E. (1998). Cross-Reactivity and Heterologous
Neutralization of Crotalinae Antivenoms Used in Argentina. Toxicon.

[34] Otero-Patiño R., Núñez V., Barona J., Díaz A., Saldarriaga M. (2002). Características bioquímicas y capacidad
neutralizante de cuatro antivenenos polivalentes frente a los efectos
farmacológicos y enzimáticos del veneno de *Bothrops
asper* y *Porthidium nasutum* de Antioquia
y Chocó. Iatreia.

[35] Rojas E., Quesada L., Arce V., Lomonte B., Rojas G., Gutiérrez J. M. (2005). Neutralization of Four Peruvian *Bothrops* sp. Snake Venoms by Polyvalent Antivenoms Produced
in Perú
and Costa Rica: Preclinical Assessment. Acta
Trop..

[36] Queiroz G. P., Pessoa L. A., Portaro F. C. V., Furtado M. de F. D., Tambourgi D. V. (2008). Interspecific
Variation in Venom Composition and Toxicity
of Brazilian Snakes from *Bothrops* Genus. Toxicon.

[37] Gutiérrez, M. , José . Snakebite Envenomation in Central America. 2009.491, 507, 10.1201/9781420008661.ch24,

[38] Segura A., Castillo M. C., Núñez V., Yarlequé A., Gonçalves L. R.
C., Villalta M., Bonilla C., Herrera M., Vargas M., Fernández M., Yano M. Y., Araújo H. P., Boller M. A. A., León P., Tintaya B., Sano-Martins I. S., Gómez A., Fernández G. P., Geoghegan P., Higashi H. G., León G., Gutiérrez J. M. (2010). Preclinical Assessment of the Neutralizing Capacity
of Antivenoms Produced in Six Latin American Countries against Medically-Relevant *Bothrops* Snake Venoms. Toxicon.

[39] Dias
da Silva W., Tambourgi D. V. (2011). Comment on Preclinical Assessment
of the Neutralizing Capacity of Antivenoms Produced in Six Latin American
Countries against Medically-Relevant *Bothrops* Snake
Venoms. Toxicon.

[40] Sousa L. F., Nicolau C. A., Peixoto P. S., Bernardoni J. L., Oliveira S. S., Portes-Junior J. A., Mourão R. H. V., Lima-dos-Santos I., Sano-Martins I. S., Chalkidis H. M., Valente R. H., Moura-da-Silva A. M. (2013). Comparison
of Phylogeny, Venom Composition and Neutralization by Antivenom in
Diverse Species of *Bothrops* Complex. PLoS Neglected Trop. Dis..

[41] De
Roodt A. R., Clement H., Dolab J. A., Litwin S., Hajos S. E., Boyer L., Alagón A. (2014). Protein Content
of Antivenoms and Relationship with Their Immunochemical Reactivity
and Neutralization Assays. Clin Toxicol..

[42] Mora-Obando D., Pla D., Lomonte B., Guerrero-Vargas J. A., Ayerbe S., Calvete J. J. (2021). Antivenomics
and *in Vivo* Preclinical Efficacy of Six Latin American
Antivenoms towards Southwestern Colombian *Bothrops asper* Lineage Venoms. PLoS Neglected Trop. Dis..

[43] Machado
Braga J. R., de Morais-Zani K., Pereira D., Sant’Anna S. S., da Costa Galizio N., Tanaka-Azevedo A. M., Gomes Vilarinho A. R., Rodrigues J. L., Teixeira da Rocha M.
M., Teixeira
da Rocha M. M. (2020). Sexual and Ontogenetic Variation of *Bothrops
leucurus* Venom. Toxicon.

[44] da
Silva Aguiar W., da Costa Galizio N., Sant’Anna S. S., Silveira G. P. M., de Souza Rodrigues F., Grego K. F., de Morais-Zani K., Tanaka-Azevedo A. M. (2020). Ontogenetic Study of *Bothrops jararacussu* Venom Composition Reveals Distinct Profiles. Toxicon.

[45] Saldarriaga M. M., Otero R., Núñez V., Toro M. F., Díaz A., Gutiérrez J. M. (2003). Ontogenetic
Variability of *Bothrops atrox* and *Bothrops
asper* Snake Venoms from Colombia. Toxicon.

[46] Madrigal M., Sanz L., Flores-Díaz M., Sasa M., Núñez V., Alape-Girón A., Calvete J. J. (2012). Snake Venomics across Genus *Lachesis*. Ontogenetic Changes in the Venom Composition of *Lachesis
stenophrys* and Comparative Proteomics of the Venoms
of Adult *Lachesis melanocephala* and *Lachesis
acrochorda*. J. Proteomics.

[47] Hatakeyama D. M., Tasima L. J., da Costa Galizio N., Serino-Silva C., Rodrigues C. F. B., Stuginski D. R., Sant’Anna S. S., Grego K. F., Tashima A. K., Nishiduka E. S., de Morais-Zani K., Tanaka-Azevedo A. M. (2021). From Birth to Adulthood: An Analysis
of the Brazilian Lancehead (*Bothrops moojeni*) Venom
at Different Life Stages. PLoS One.

[48] De-Oliveira C. A., Stuginski D. R., Kitano E. S., Andrade-Silva D., Liberato T., Fukushima I., Serrano S. M. T., Zelanis A. (2016). Dynamic Rearrangement
in Snake Venom Gland Proteome: Insights into *Bothrops jararaca* Intraspecific Venom Variation. J. Proteome
Res..

[49] Pimenta D. C., Prezoto B. C., Konno K., Melo R. L., Furtado M. F., Camargo A. C. M., Serrano S. M. T. (2007). Mass
Spectrometric Analysis of the
Individual Variability of *Bothrops jararaca* Venom
Peptide Fraction. Evidence for Sex-Based Variation among the Bradykinin-Potentiating
Peptides. Rapid Commun. Mass Spectrom..

[50] Huang H. W., Liu B. S., Chien K. Y., Chiang L. C., Huang S. Y., Sung W. C., Wu W. G. (2015). Cobra Venom
Proteome and Glycome
Determined from Individual Snakes of *Naja atra* Reveal
Medically Important Dynamic Range and Systematic Geographic Variation. J. Proteomics.

[51] Sousa L. F., Portes-Junior J. A., Nicolau C. A., Bernardoni J. L., Nishiyama-Jr M. Y., Amazonas D. R., Freitas-de-Sousa L. A., Mourão R. H., Chalkidis H. M., Valente R. H., Moura-da-Silva A. M. (2017). Functional
Proteomic Analyses of *Bothrops atrox* Venom Reveals
Phenotypes Associated with Habitat Variation in the Amazon. J. Proteomics.

[52] Zaher H., Murphy R. W., Arredondo J. C., Graboski R., Machado-Filho P. R., Mahlow K., Montingelli G. G., Quadros A. B., Orlov N. L., Wilkinson M., Zhang Y. P., Grazziotin F. G. (2019). Large-Scale
Molecular Phylogeny, Morphology, Divergence-Time Estimation, and the
Fossil Record of Advanced Caenophidian Snakes (Squamata: Serpentes). PLoS One.

[53] Grego K. F., Vieira S. E. M., Vidueiros J. P., Serapicos E. d. O., Barbarini C. C., Silveira G. P. M. da., Rodrigues F. d. S., Alves L. d. C. F., Stuginski D. R., Rameh-de-Albuquerque L. C., Furtado M. d. F. D., Tanaka-Azevedo A. M., Morais-Zani K. de., Rocha M. M. T. da., Fernandes W., Sant’Anna S. S. (2021). Maintenance of Venomous Snakes in
Captivity for Venom Production at Butantan Institute from 1908 to
the Present: A Scoping History. J. Venom. Anim.
Toxins Incl. Trop. Dis..

[54] Bradford M. M. (1976). Rapid and
Sensitive Method for Quantitation of Microgram Quantities of Protein
Utilizing Principle of Protein-Dye Binding. Anal. Biochem..

[55] Laemmli U. K. (1970). Cleavage
of Structural Proteins during the Assembly of the Head of Bacteriophage
T4. Nature.

[56] Eichberg S., Sanz L., Calvete J. J., Pla D. (2015). Constructing Comprehensive
Venom Proteome Reference Maps for Integrative Venomics. Expert Rev. Proteomics.

[57] Calderón-Celis F., Cid-Barrio L., Encinar J. R., Sanz-Medel A., Calvete J. J. (2017). Absolute Venomics:
Absolute Quantification of Intact
Venom Proteins through Elemental Mass Spectrometry. J. Proteomics.

[58] Deutsch E. W., Bandeira N., Perez-Riverol Y., Sharma V., Carver J. J., Mendoza L., Kundu D. J., Wang S., Bandla C., Kamatchinathan S., Hewapathirana S., Pullman B. S., Wertz J., Sun Z., Kawano S., Okuda S., Watanabe Y., Maclean B., Maccoss M. J., Zhu Y., Ishihama Y., Vizcaíno J. A. (2023). The ProteomeXchange
Consortium at 10 Years: 2023 Update. Nucleic
Acids Res..

[59] Perez-Riverol Y., Bandla C., Kundu D. J., Kamatchinathan S., Bai J., Hewapathirana S., John N. S., Prakash A., Walzer M., Wang S., Vizcaíno J. A. (2025). The PRIDE
Database at 20 Years: 2025 Update. Nucleic Acids
Res..

[60] Váchová L., Moravcová J. (1993). Two Microassays for Determination of a Wide Range of
Proteolytic Activities Using Azocoll as Substrate. Biochem Mol. Biol. Int..

[61] Antunes T. C., Yamashita K. M., Barbaro K. C., Saiki M., Santoro M. L. (2010). Comparative
Analysis of Newborn and Adult *Bothrops jararaca* Snake
Venoms. Toxicon.

[62] Mackessy P., Holzer M. (1996). An Aqueous Endpoint Assay of Snake
Venom Phospholipase
A_2_. Toxicon.

[63] CIOMS . International Guiding Principles for Biomedical Research Involving Animals, 1985. 11653736

[64] Kilkenny C., Browne W. J., Cuthill I. C., Emerson M., Altman D. G. (2010). Improving
Bioscience Research Reporting: The ARRIVE Guidelines for Reporting
Animal Research. J. Pharmacol. Pharmacother..

[65] Finney, D. J. Probit Analysis: 3d Ed; Cambridge University Press, 1971.

[66] Kondo H., Kondo S., Kezawa H., Murata R., Ohsaka A. (1960). Studies on
the Quantitative Method for Determination of Hemorrhagic Activity
of Habu Snake Venom. Jpn. J. Med. Sci. Biol..

[67] Gutiérrez J., Gené J., Rojas G., Cerdas L. (1985). Neutralization of Proteolytic
and Hemorrhagic Activities of Costa Rican Snake Venoms by a Polyvalent
Antivenom. Toxicon.

[68] Levy L. (1969). Carrageenan
Paw Edema in the Mouse. Life Sci..

[69] Segura Á., Herrera M., Mares F. R., Jaime C., Sánchez A., Vargas M., Villalta M., Gómez A., Gutiérrez J. M., León G. (2017). Proteomic
Toxicological and Immunogenic
Characterization of Mexican West-Coast Rattlesnake (*Crotalus
basiliscus*) Venom and Its Immunological Relatedness with
the Venom of Central American Rattlesnake (*Crotalus simus*). J. Proteomics.

[70] Gutiérrez J. M., Alberto Ponce-Soto L., Marangoni S., Lomonte B. (2008). Systemic and Local
Myotoxicity Induced by Snake Venom Group II Phospholipases A_2_: Comparison between Crotoxin, Crotoxin B and a Lys49 PLA_2_ Homologue. Toxicon.

[71] Pla D., Rodríguez Y., Calvete J. J. (2017). Third Generation Antivenomics: Pushing
the Limits of the in Vitro Preclinical Assessment of Antivenoms. Toxins.

[72] Calvete J. J., Rodríguez Y., Quesada-Bernat S., Pla D. (2018). Toxin-Resolved Antivenomics-Guided
Assessment of the Immunorecognition Landscape of Antivenoms. Toxicon.

[73] Howard, G. C. ; Kaser, M. R. Making and Using Antibodies: A Practical Handbook, Second edi ed.; CRC Press, Taylor & Francis Group: Boca Raton (FL), 2014.

[74] Araujo H. P., Bourguignon S. C., Boller M. A. A., Dias A. A. S. O., Lucas E. P. R., Santos I. C., Delgado I. F. (2008). Potency Evaluation
of Antivenoms in Brazil: The National Control Laboratory Experience
between 2000 and 2006. Toxicon.

[75] Morais V., Ifran S., Berasain P., Massaldi H. (2010). Antivenoms: Potency
or Median Effective Dose, Which to Use?. J.
Venom. Anim. Toxins incl. Trop. Dis. V.

[76] Gutiérrez J. M., Rucavado A., Escalante T., Díaz C. (2005). Hemorrhage
Induced by Snake Venom Metalloproteinases: Biochemical and Biophysical
Mechanisms Involved in Microvessel Damage. Toxicon.

[77] Gutiérrez, J. M. ; Escalante, T. ; Rucavado, A. ; Herrera, C. ; Fox, J. W. A Comprehensive View of the Structural and Functional Alterations of Extracellular Matrix by Snake Venom Metalloproteinases (SVMPs): Novel Perspectives on the Pathophysiology of Envenoming. Toxins. MDPI AG October 1, 2016. 10.3390/toxins8100304 PMC5086664 27782073

[78] Albuquerque P. L. M.
M., Paiva J. H. H. G. L., Martins A. M. C., Meneses G. C., Silva
Júnior G. B. d., Buckley N., Daher E. D. F. (2020). Clinical
Assessment and Pathophysiology of *Bothrops* Venom-Related
Acute Kidney Injury: A Scoping Review. J. Venom.
Anim. Toxins Incl. Trop. Dis..

[79] Vidal J. F. D., Schwartz M. F., Garay A. V., Valadares N. F., Bueno R. V., Monteiro A. C. L., Freitas S. M. de., Barbosa J. A. R. G. (2024). Exploring
the Diversity and Function of Serine Proteases in Toxicofera Reptile
Venoms: A Comprehensive Overview. Toxins.

[80] Fernandes C. A. H., Borges R. J., Lomonte B., Fontes M. R. M. (2014). A Structure-Based
Proposal for a Comprehensive Myotoxic Mechanism of Phospholipase A_2_-like Proteins from Viperid Snake Venoms. Biochim. Biophys. Acta, Proteins Proteomics.

[81] Mora-Obando D., Fernández J., Montecucco C., Gutiérrez J. M., Lomonte B. (2014). Synergism between Basic Asp49 and
Lys49 Phospholipase
A_2_ Myotoxins of Viperid Snake Venom *in Vitro* and *in Vivo*. PLoS One.

[82] Fry, B. Venomous Reptiles and their Toxins: Evolution, Pathophysiology and Biodiscovery; Oxford University Press, 2015.

[83] James
Graham R. L., Graham C., McClean S., Chen T., O’Rourke M., Hirst D., Theakston D., Shaw C. (2005). Identification and Functional Analysis of a Novel Bradykinin Inhibitory
Peptide in the Venoms of New World Crotalinae Pit Vipers. Biochem. Biophys. Res. Commun..

[84] Cidade D. A. P., Simão T. A., Dávila A. M. R., Wagner G., de L M Junqueira-de-Azevedo I., Lee Ho P., Bon C., Zingali R. B., Albano R. M., Albano R. M. (2006). *Bothrops jararaca* Venom Gland Transcriptome:
Analysis of the Gene Expression Pattern. Toxicon.

[85] Huang K.-F., Hung C.-C., Wu S.-H., Chiou S.-H. (1998). Characterization
of Three Endogenous Peptide Inhibitors for Multiple Metalloproteinases
with Fibrinogenolytic Activity from the Venom of Taiwan Habu (*Trimeresurus mucrosquamatus*). Biochem.
Biophys. Res. Commun..

[86] Huang K. F., Chiou S. H., Ko T. P., Wang A. H. J. (2002). Determinants
of the Inhibition of a Taiwan Habu Venom Metalloproteinase by Its
Endogenous Inhibitors Revealed by X-Ray Crystallography and Synthetic
Inhibitor Analogues. Eur. J. Biochem..

[87] Kato H., Iwanaga S., Suzuki T. (1966). The Isolation
and Amino Acid Sequences
of New Pyroglutamylpeptides from Snake Venoms. Experientia.

[88] Munekiyo S. M., Mackessy S. P. (2005). Presence of Peptide
Inhibitors in Rattlesnake Venoms
and Their Effects on Endogenous Metalloproteases. Toxicon.

[89] Wagstaff S. C., Favreau P., Cheneval O., Laing G. D., Wilkinson M. C., Miller R. L., Stöcklin R., Harrison R. A. (2008). Molecular Characterisation
of Endogenous Snake Venom Metalloproteinase Inhibitors. Biochem. Biophys. Res. Commun..

[90] Mora-Obando D., Salazar-Valenzuela D., Pla D., Lomonte B., Guerrero-Vargas J. A., Ayerbe S., Gibbs H. L., Calvete J. J. (2020). Venom Variation
in *Bothrops asper* Lineages from North-Western South
America. J. Proteomics.

[91] Valdujo P. H., Nogueira C., Martins M. (2002). Ecology of *Bothrops neuwiedi
pauloensis* (Serpentes: Viperidae: Crotalinae) in the Brazilian
Cerrado. J. Herpetol..

[92] de Oliveira, E. T. Biologia alimentar e reprodutiva da jararaca-pintada Bothrops diporus (Serpentes; Viperidae) na Mata Atlântica de interior, Sul do Brasil; Universidade Comunitária da Região de Chapecó: Chapecó, 2015.

[93] Hartmann M.
T., Hartmann P. A., Cechin S. Z., Martins M. (2005). Feeding Habits and
Habitat Use in *Bothrops pubescens* (Viperidae, Crotalinae)
from Southern Brazil. J. Herpetol..

[94] Martins, M. ; Marques, O. A. V. ; Sazima, I. Ecological and Phylogenetic Correlates of Feeding Habits in Neotropical Pitvipers of the Genus *Bothrops* . In Biology of the Vipers; Schuett, G. W. , Hoggren, M. , Douglas, M. E. , Eds.; Eagle Mountain Pub Lc, 2002; pp 307–328.

[95] Monteiro C., Montgomery C. E., Spina F., Sawaya R. J., Martins M. (2006). Feeding, Reproduction,
and Morphology of *Bothrops mattogrossensis* (Serpentes,
Viperidae, Crotalinae) in the Brazilian Pantanal. J. Herpetol..

[96] Gutiérrez J. M., Lomonte B. (1995). Local Pathological
Effects Induced by *Bothrops* Snake Venoms. Mem. Inst. Butantan.

[97] De
Andrade Nishioka S., Silveira P. V. P. (1992). A Clinical and Epidemiologic Study
of 292 Cases of Lance-Headed Viper Bite in a Brazilian Teaching Hospital. Am. J. Trop. Med. Hyg..

[98] Warrell D. A. (2004). Snakebites
in Central and South America: Epidemiology, Clinical Features, and
Clinical Management. Venomous Reptiles Western
Hemisphere.

[99] Jorge M. T., Ribeiro L. A. (2000). Envenoming by the
South American Pit Viper *Bothrops neuwiedi* Wagler. Ann. Trop.
Med. Parasitol..

[100] Dempfle E., Kohl R., Harenberg J., Kirschstein W., Schlauch D., Heene D. (1990). Coagulopathy after
Snake Bite by *Bothrops neuwiedi*: Case Report and
Results of *in Vitro* Experiments. Blut.

[101] Marinho A. D., Silveira J. A. d. M., Chaves Filho A. J. M., Jorge A. R. C., Nogueira Júnior F. A., Pereira V. B. M., de Aquino P. E. A., Pereira C. A. S., Evangelista J. S. A. M., Macedo D. S. (2021). *Bothrops pauloensis* Snake
Venom-Derived Asp-49 and Lys-49 Phospholipases A_2_ Mediates
Acute Kidney Injury by Oxidative Stress and Release of Inflammatory
Cytokines. Toxicon.

[102] Escalante T., Rucavado A., Fox J. W., Gutiérrez J. M. (2011). Key Events
in Microvascular Damage Induced by Snake Venom Hemorrhagic Metalloproteinases. J. Proteomics.

[103] Gutiérrez J., Lomonte B. (1995). Phospholipase A_2_ Myotoxins
from *Bothrops* Snake Venoms. Toxicon.

[104] Gutiérrez J. M., Ownby C. L. (2003). Skeletal Muscle Degeneration Induced
by Venom Phospholipases A_2_: Insights into the Mechanisms
of Local and Systemic Myotoxicity. Toxicon.

[105] Lomonte B., Angulo Y., Sasa M., Gutierrez J. (2009). The Phospholipase
A_2_ Homologues of Snake Venoms: Biological Activities and
Their Possible Adaptive Roles. Protein Pept.
Lett..

[106] Daniele J. J., Bianco D., Delgado C., Briones
Carrillo D., Fidelio G. D., Bianco I. D., Delgado
Briones Carrillo C. D. (1997). A New Phospholipase A_2_ Isoform Isolated
from *Bothrops neuwiedi* (Yarará Chica) Venom
with Novel Kinetic and Chromatographic Properties. Toxicon.

[107] Geoghegan P., Angulo Y., Cangelosi A., Nica Dõâ Az M. A. ^., Lomonte B. (1999). Characterization of
a Basic Phospholipase A_2_-Homologue Myotoxin Isolated from
the Venom of the Snake *Bothrops neuwiedii* (Yarara
Chica) from Argentina. Toxicon.

[108] Markland F. S. (1998). Snake Venom
and Hemostatic System. Toxicon.

[109] Kini R. M. (2006). Anticoagulant Proteins from Snake
Venoms: Structure,
Function and Mechanism. Biochem. J..

[110] Kini R. M., Rao V. S., Joseph J. S. (2002). Procoagulant
Proteins
from Snake Venoms. Haemostasis.

[111] Isbister G. K. (2009). Procoagulant
Snake Toxins: Laboratory Studies, Diagnosis,
and Understanding Snakebite Coagulopathy. Semin.
Thromb. Hemost..

[112] Calvete J. J., Marcinkiewicz C., Monleón D., Esteve V., Celda B., Juárez P., Sanz L. (2005). Snake Venom Disintegrins: Evolution of Structure and Function. Toxicon.

[113] Arlinghaus F. T., Eble J. A. (2012). C-Type Lectin-like
Proteins from
Snake Venoms. Toxicon.

[114] Yamazaki Y., Takani K., Atoda H., Morita T. (2003). Snake Venom
Vascular Endothelial Growth Factors (VEGFs) Exhibit Potent Activity
through Their Specific Recognition of KDR (VEGF Receptor 2). J. Biol. Chem..

[115] Yamazaki Y., Nakano Y., Imamura T., Morita T. (2007). Augmentation
of Vascular Permeability of VEGF Is Enhanced by KDR-Binding Proteins. Biochem. Biophys. Res. Commun..

[116] Ferreira S. H., Bartelt D. C., Greene L. J. (1970). Isolation of Bradykinin-Potentiating
Peptides from *Bothrops jararaca* Venom. Biochemistry.

[117] Greene L. J., Em K., Sh F. (1972). Inhibition
of the Conversion
of Angiotensin I to II and Potentiation of Bradykinin by Small Peptides
Present in *Bothrops jararaca* Venom. Circ. Res..

[118] Luft F. C. (2008). The *Bothrops Legacy*: Vasoactive Peptides
from Brazil. J. Renin-Angiotensin-Aldosterone
Syst. JRAAS.

[119] Galizio N. da C., Serino-Silva C., Stuginski D. R., Abreu P. A. E., Sant’Anna S. S., Grego K. F., Tashima A. K., Tanaka-Azevedo A. M., Morais-Zani K. de. (2018). Compositional and Functional Investigation
of Individual and Pooled Venoms from Long-Term Captive and Recently
Wild-Caught *Bothrops jararaca* Snakes. J. Proteomics.

[120] Aguiar W. d. S., Galizio N. d. C., Serino-Silva C., Sant’Anna S. S., Grego K. F., Tashima A. K., Nishiduka E. S., Morais-Zani K. d., Tanaka-Azevedo A. M. (2019). Comparative Compositional and Functional
Analyses of *Bothrops moojeni* Specimens Reveal Several
Individual Variations. PLoS One.

[121] Herrera C., Escalante T., Voisin M.-B., Rucavado A., Morazán D., Macêdo J. K. A., Calvete J. J., Sanz L., Nourshargh S., Gutiérrez J. M., Fox J. W. (2015). Tissue Localization
and Extracellular Matrix Degradation by PI, PII and PIII Snake Venom
Metalloproteinases: Clues on the Mechanisms of Venom-Induced Hemorrhage. PLoS Neglected Trop. Dis..

[122] Moura-Da-Silva A. M., Cardoso D. F., Tanizaki M. M. (1990). Differences
in Distribution
of Myotoxic Proteins in Venoms from Different *Bothrops* Species. Toxicon.

[123] Zamunér S. R., da Cruz-Höfling M. A., Corrado A. P., Hyslop S., Rodrigues-Simioni L. (2004). Comparison
of the Neurotoxic and
Myotoxic Effects of Brazilian *Bothrops* Venoms and
Their Neutralization by Commercial Antivenom. Toxicon.

[124] Moura A. A. D., Kayano A. M., Oliveira G. A., Setúbal S. S., Ribeiro J. G., Barros N. B., Nicolete R., Moura L. A., Fuly A. L., Nomizo A., da Silva S. L., Fernandes C. F. C., Zuliani J. P., Stábeli R. G., Soares A. M., Calderon L. A. (2014). Purification
and Biochemical Characterization of Three Myotoxins from *Bothrops
mattogrossensis* Snake Venom with Toxicity against *Leishmania* and Tumor Cells. Biomed.
Res. Int..

[125] Bustillo S., Fernández J., Chaves-Araya S., Angulo Y., Leiva L. C., Lomonte B. (2019). Isolation of Two Basic
Phospholipases A_2_ from *Bothrops diporus* Snake Venom: Comparative Characterization and Synergism between
Asp49 and Lys49 Variants. Toxicon.

[126] Teixera L. F., de Carvalho L. H., de Castro O. B., Bastos J. S. F., Néry N. M., Oliveira G. A., Kayano A. M., Soares A. M., Zuliani J. P. (2018). Local and
Systemic Effects of BdipTX-I,
a Lys-49 Phospholipase A_2_ Isolated from *Bothrops
diporus* Snake Venom. Toxicon.

[127] Mora-Obando D., Fernández J., Montecucco C., Gutiérrez J. M., Lomonte B. (2014). Synergism between Basic
Asp49 and
Lys49 Phospholipase A_2_ Myotoxins of Viperid Snake Venom *in Vitro* and *in Vivo*. PLoS One.

[128] Moreira V., De Castro Souto P. C.
M., Ramirez Vinolo M. A., Lomonte B., María Gutiérrez J., Curi R., Teixeira C. (2013). A Catalytically-Inactive Snake Venom
Lys49 Phospholipase A_2_ Homolog Induces Expression of Cyclooxygenase-2
and Production of Prostaglandins through Selected Signaling Pathways
in Macrophages. Eur. J. Pharmacol..

[129] Teixeira C., Cury Y., Moreira V., Picolo G., Chaves F. (2009). Inflammation Induced by *Bothrops
asper* Venom. Toxicon.

[130] Lomonte, B. Lys49 Myotoxins, Secreted Phospholipase A_2_-like Proteins of Viperid Venoms: A Comprehensive Review. Toxicon. Elsevier Ltd March 1, 2023. DOI: 10.1016/j.toxicon.2023.107024. 36632869

[131] Nery N. M., Luna K. P., Fernandes C. F. C., Zuliani J. P. (2016). An Overview of Bothrops erythromelas Venom. Rev. Soc. Bras. Med. Trop..

[132] Nadur-Andrade N., Barbosa A. M., Carlos F. P., Lima C. J., Cogo J. C., Zamuner S. R. (2012). Effects of Photobiostimulation
on
Edema and Hemorrhage Induced by *Bothrops moojeni* Venom. Lasers Med. Sci..

[133] Chaves F., Barboza M., Gutiérrez J. M. (1995). Pharmacological
Study of Edema Induced by Venom of the Snake *Bothrops asper* (Terciopelo) in Mice. Toxicon.

[134] Santoro M. L., Sano-Martins I. S., Fan H. W., Cardoso J. L., Theakston R. D. G., Warrell D. A. (2008). Haematological Evaluation of Patients
Bitten by the Jararaca, *Bothrops jararaca*, in Brazil. Toxicon.

[135] Lotto N. P., de Albuquerque Modesto J. C., Sant’Anna S. S., Grego K. F., Guarnieri M. C., Lira-Da-Silva R. M., Santoro M. L., Oguiura N. (2021). The Absence of Thrombin-like Activity
in *Bothrops erythromelas* Venom Is Due to the Deletion
of the Snake Venom Thrombin-like Enzyme Gene. PLoS One.

[136] Larréché S., Chippaux J. P., Chevillard L., Mathé S., Résière D., Siguret V., Mégarbane B. (2021). Bleeding and
Thrombosis: Insights into Pathophysiology
of *Bothrops* Venom-Related Hemostasis Disorders. Int. J. Mol. Sci..

[137] Nahas L., Kamiguti A. S., Barros M. A. R. (1979). Thrombin-Like
and Factor X-Activator Components of *Bothrops* Snake
Venoms. Thromb. Haemost..

[138] Furtado M. F. D., Maruyama M., Kamiguti A. S., Antonio L. C. (1991). Comparative
Study of Nine *Bothrops* Snake Venoms from Adult Female
Snakes and Their Offspring. Toxicon.

[139] Hatakeyama D. M., Tasima L. J., Bravo-Tobar C. A., Serino-Silva C., Tashima A. K., Rodrigues C. F. B., Aguiar W. d. S., Galizio N. d. C., Lima E. O. V. d., Kavazoi V. K., Gutierrez-Marín J. D., Farias I. B. d., Sant’Anna S. S., Grego K. F., Morais-Zani K. d., Tanaka-Azevedo A. M. (2020). Venom Complexity of *Bothrops atrox* (Common Lancehead) Siblings. J. Venom. Anim.
Toxins Incl. Trop. Dis..

[140] Chacón F., Oviedo A., Escalante T., Solano G., Rucavado A., Gutiérrez J. M. (2015). The Lethality
Test Used for Estimating the Potency of Antivenoms against *Bothrops asper* Snake Venom: Pathophysiological Mechanisms,
Prophylactic Analgesia, and a Surrogate *in Vitro* Assay. Toxicon.

[141] Ferreira M. L., Moura-da-Silva A. M., França F. O. S., Cardoso J. L., Mota I. (1992). Toxic Activities of
Venoms from Nine *Bothrops* Species and Their Correlation
with Lethality and
Necrosis. Toxicon.

[142] Mackessy S. P. (2010). Evolutionary Trends in Venom Composition
in the Western
Rattlesnakes (*Crotalus viridis* Sensu Lato): Toxicity
vs. Tenderizers. Toxicon.

[143] Mackessy S. P. (2008). Venom Composition in Rattlesnakes:
Trends and Biological
Significance. Biol. Rattlesnakes.

[144] Ferreira M. L., Moura-da-Silva A. M., Mota I. (1992). Neutralization of Different
Activities of Venoms from Nine Species of *Bothrops* Snakes by *Bothrops jararaca* Antivenom. Toxicon.

[145] Bogarín G., Morais J. F., Yamaguchi I. K., Stephano M. A., Marcelino J. R., Nishikawa A. K., Guidolin R., Rojas G., Higashi H. G., Gutiérrez J. M., Gondo Higashi H., Marõâ A
Gutieâ Rrez J. (2000). Neutralization
of Crotaline Snake Venoms from Central and South America by Antivenoms
Produced in Brazil and Costa Rica. Toxicon.

[146] Sanz L., Pérez A., Quesada-Bernat S., Diniz-Sousa R., Calderón L. A., Soares A. M., Calvete J. J., Caldeira C. A. S. (2020). Venomics and
Antivenomics of the Poorly Studied Brazil’s
Lancehead, *Bothrops brazili* (Hoge, 1954), from the
Brazilian State of Pará. *Journal of Venomous Animals
and Toxins Including Tropical Diseases*. J. Venom Anim. Toxins Incl. Trop. Dis..

[147] Muniz E. G., Sano-Martins I. S., Saraiva M., Monteiro W. M., Magno E. S., Oliveira S. S., Oliveira S. S. (2023). Biological Characterization
of the *Bothrops brazili* Snake Venom and Its Neutralization
by Brazilian *Bothrops* Antivenom Produced by the Butantan
Institute. Toxicon.

[148] Félix-Silva J., Gomes J. A. S., Xavier-Santos J. B., Passos J. G. R., Silva-Junior A. A., Tambourgi D. V., Fernandes-Pedrosa M.
F. (2017). Inhibition of Local
Effects Induced by *Bothrops
erythromelas* Snake Venom: Assessment of the Effectiveness
of Brazilian Polyvalent Bothropic Antivenom and Aqueous Leaf Extract
of *Jatropha gossypiifolia*. Toxicon.

[149] Boechat A. L. R., Paiva C. S., França F. O., Dos-Santos M. C. (2001). Heparin-Antivenom Association: Dfferential Neutralization
Effectiveness in *Bothrops atrox* and *Bothrops
erythromelas* Envenoming. Rev. Inst.
Med. trop. S. Paulo.

[150] Valente R. H., Guimarães P. R., Junqueira M., Neves-Ferreira A. G. C., Soares M. R., Chapeaurouge A., Trugilho M. R. O., León I. R., Rocha S. L. G., Oliveira-Carvalho A.
L., Wermelinger L. S., Dutra D. L. S., Leão L. I., Junqueira-de-Azevedo I. L. M., Ho P. L., Zingali R. B., Perales J., Domont G. B. (2009). *Bothrops insularis*: Venomics: A Proteomic Analysis Supported
by Transcriptomic-Generated
Sequence Data. J. Proteomics.

[151] Andrade-Silva D., Zelanis A., Travaglia-Cardoso S. R., Nishiyama M. Y., Serrano S. M. T. (2021). Venom Profiling of the Insular Species *Bothrops alcatraz*: Characterization of Proteome, Glycoproteome,
and N-Terminome Using Terminal Amine Isotopic Labeling of Substrates. J. Proteome Res..

[152] Chiarelli T., Hayashi J. Y., Galizio N. da C., Casimiro F. M. S., Torquato R., Tanaka A. S., Morais-Zani K. de, Tanaka-Azevedo A. M., Tashima A. K. (2025). Enhancing the Bothropic Antivenom
through a Reverse Antivenomics Approach. J.
Proteome Res..

